# Microkinetic Analysis of the Oxygen Evolution Performance at Different Stages of Iridium Oxide Degradation

**DOI:** 10.1021/jacs.2c03561

**Published:** 2022-07-18

**Authors:** Janis Geppert, Philipp Röse, Steffen Czioska, Daniel Escalera-López, Alexey Boubnov, Erisa Saraçi, Serhiy Cherevko, Jan-Dierk Grunwaldt, Ulrike Krewer

**Affiliations:** † Institute for Applied Materials-Electrochemical Technologies (IAM-ET), Karlsruhe Institute of Technology, Adenauerring 20b, Karlsruhe 76131, Germany; ‡ Institute for Chemical Technology and Polymer Chemistry (ITCP), Karlsruhe Institute of Technology, Engesserstr. 20, Karlsruhe 76131, Germany; § Helmholtz-Institute Erlangen-Nürnberg for Renewable Energy (IEK-11), Forschungszentrum Jülich GmbH, Cauerstr. 1, Erlangen 91058, Germany; ∥ Institute of Catalysis Reasearch and Technology (IKFT), Karlsruhe Institute of Technology, Hermann-von-Helmholtz-Platz 1, Eggenstein-Leopoldshafen 76344, Germany

## Abstract

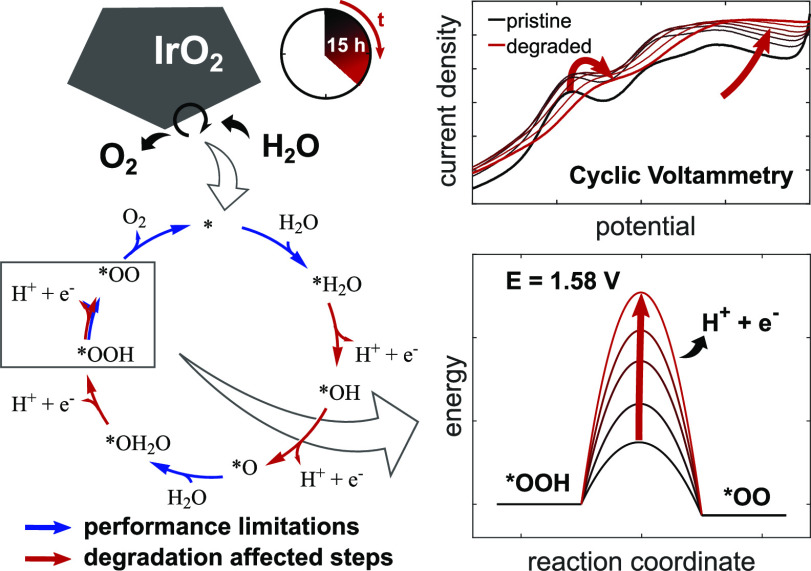

The microkinetics of the electrocatalytic oxygen evolution reaction substantially determines the performance in proton-exchange membrane water electrolysis. State-of-the-art nanoparticulated rutile IrO_2_ electrocatalysts present an excellent trade-off between activity and stability due to the efficient formation of intermediate surface species. To reveal and analyze the interaction of individual surface processes, a detailed dynamic microkinetic model approach is established and validated using cyclic voltammetry. We show that the interaction of three different processes, which are the adsorption of water, one potential-driven deprotonation step, and the detachment of oxygen, limits the overall reaction turnover. During the reaction, the active IrO_2_ surface is covered mainly by *O, *OOH, and *OO adsorbed species with a share dependent on the applied potential and of 44, 28, and 20% at an overpotential of 350 mV, respectively. In contrast to state-of-the-art calculations of ideal catalyst surfaces, this novel model-based methodology allows for experimental identification of the microkinetics as well as thermodynamic energy values of real pristine and degraded nanoparticles. We show that the loss in electrocatalytic activity during degradation is correlated to an increase in the activation energy of deprotonation processes, whereas reaction energies were marginally affected. As the effect of electrolyte-related parameters does not cause such a decrease, the model-based analysis demonstrates that material changes trigger the performance loss. These insights into the degradation of IrO_2_ and its effect on the surface processes provide the basis for a deeper understanding of degrading active sites for the optimization of the oxygen evolution performance.

## 1 Introduction

Polymer electrolyte membrane (PEM) water electrolysis is one of the key technologies in a sustainable energy system based on renewable resources.^1,2^ To provide a high overall electrolysis performance, costly but electrocatalytically active and stable materials are required at both electrode processes: while the cathodic hydrogen evolution reaction (2H^+^ + 2e^–^ → H_2_) is efficiently catalyzed using Pt,^3^ its anodic counterpart, the oxygen evolution reaction (OER, 2H_2_O → 4H^+^ + 4e^–^ + O_2_), is heavily limited by the sluggish and complex microkinetics.^4^ Multiple OER catalysts have been screened over the last decade. Among them, IrO_2_ was found to outperform most of the active transition metals and their oxides, and it provides the highest stability under harsh process conditions with strong oxidizing potentials in acidic media.^5,6^ Therefore, it is considered as a benchmark material in PEM water electrolysis.

In a PEM cell assembly operated at 3 A cm^–2^, only minor overpotentials are induced by hydrogen mass transport (∼20 mV) and proton conduction resistance (∼20 mV), and the Ohmic losses are reported to account for 155 mV.^4^ The major loss arises due to the OER kinetics, which is even for highly active IrO_2_ quantified with an overpotential of about ∼350 mV in the PEM cell assembly.^4^ An in-depth understanding of the microkinetics at the surface and the kinetically and thermodynamically limiting processes is, thus, of major interest to optimize the catalytic system. Moreover, high operation potentials are applied to reach technically relevant conversion rates. These provoke side reactions and processes that lead to catalyst degradation and, as a result, a lowered activity.^7^ A recently reported process that leads to a performance decrease during operation is the formation of nano- and micro-sized oxygen bubbles in the electrolyte phase.^8^ As a degradation process, dissolution of the active IrO_2_ material was detected and quantified using a scanning flow cell coupled downstream to an inductively coupled plasma mass spectrometry (ICP–MS) system.^5,9^ The use of operando X-ray absorption spectroscopy has recently proven the formation of oxygen vacancies during the OER.^10^ Interestingly, their formation led to a stabilization of crystalline IrO_2_. The extent and way in which degradation affects the microkinetics of the IrO_2_ surface processes are still unresolved, although they account for the major impact on the catalyst’s performance. This demonstrates the urgent need to analyze and quantify activity-defining processes at the catalyst surface and their degradation-related changes under operation conditions.

The electrocatalytic activity of IrO_2_ is explained by adsorbed intermediates that pave a thermodynamically efficient pathway alongside the reaction coordinate of the OER.^11,12^ Although the free-energy values were extensively studied by density functional theory (DFT) approaches,^13,14^ the reaction energies and even the identified reaction that constitutes the overpotential defining step vary drastically with the applied computational details.^15^ To circumvent this issue, kinetic modeling approaches have been employed, in which rate equations are parametrized by Tafel slope data and DFT results to study the energy profile and the coverage of the surface at different applied potentials under steady-state conditions.^16,17^ Recent analyses suggest two different rate-determining steps depending on the applied overpotential,^18^ and a corresponding change in the charge is correlated to the surface coverage of adsorbed species.^19^

The catalytic system is highly dynamic, and evaluating the surface processes solely on the basis of steady-state experiments can result in misleading conclusions. Model-based kinetic analysis of the surface processes shows that by employing steady-state OER experiments, microkinetic parameters could not be identified.^20,21^ In contrast, combining dynamic experiments, such as cyclic voltammetry (CV), impedance spectroscopy, or chronopotentiometry/amperometry, with dynamic microkinetic modeling allows to elucidate the surface processes and effects of further chemical or transport processes and to study limitations. This has been demonstrated by revealing the reaction kinetics of acidic^22,23^ and alkaline methanol oxidation including catalyst passivation^24^ by the kinetics^25^ and poisoning^26^ of the cathode in PEM fuel cells and even for kinetics that involve chemical reactions in the electrolyte^27^ or bioelectrochemical reactions.^28^ Recently, it was applied to the OER on planar hydrous Ir to identify the kinetic rate constants and the OER mechanism by using CV curves.^29^ No work on kinetic identification based on experimental CV curves of nanoparticulate OER catalysts and on rutile IrO_2_ as the technical state-of-the-art OER catalyst is available; however, such analysis would give in-depth insight into the performance limitations and may trigger improved commercial catalysts and electrolysis. In our prior work, we used a microkinetic representation solely by rate constants. However, it does not give explicit information on essential thermodynamic energy parameters, which hampers comparison to other reactions and especially to ab initio determined energy parameters. To gain a comprehensive understanding of all relevant interactions, we here propose a fundamental physical description based on such energy values. An appropriate methodology for this purpose will be presented here for the first time.

Experimental studies on rutile structured IrO_2_, prepared by exposing it to increasing calcination temperatures, widely conclude on increasing electrocatalytic stability but decreasing activity.^9,30^ In a recent interesting work, the kinetics of the stability-related dissolution processes was modeled with a network structure approach.^31^ So far, there are no reported microkinetic studies on the effect of degradation on the OER kinetics on rutile IrO_2_ itself. However, the effect of the material degradation on the OER performance is one of the most relevant issues to address to ensure long-term stability. In general, degradation studies are rare and focus mostly on experimentally observable values such as overpotential and current density.^32^ To date, it is still unknown which interactions of surface processes define the OER performance on IrO_2_ and how they change due to operation-related degradation. In the present study, we provide a novel model-assisted microkinetic methodology for degradation analysis of electrocatalysts using CV and employ it for the first time for degradation analysis of the important OER benchmark catalyst. This allows to access and quantify all relevant degradation parameters, thus enabling a holistic understanding of the OER performance. We envision the methodology to be transferable and useful for analysis of also other electrocatalysts and reactions.

Present state-of-the-art approaches for model-based reaction analysis of OER use DFT to calculate energy values for ideal catalyst surfaces with ideal conditions, not nanoparticulate catalysts in an electrolyte. Only few microkinetic modeling studies on the OER on IrO_2_ exist, where ideal DFT values are used to predict the microkinetics and process rates.^13,16^ In contrast, our method starts at the experiment and quantifies the kinetics and thermodynamic energies from dynamic experimental data using a kinetic model. This ensures a realistic analysis of the complex situation at the nanoparticulated catalyst surface of CV experiments. The methodology was now conducted for the OER on rutile IrO_2_ for the first time.

Analyzing the drift of these parameters with time due to degradation is a natural further step we present in this article, which does not require bottom-up guessing of degradation mechanisms. The methodology can, thus, be applied to analyze catalyst surfaces during OER operation over multiple hours. It may even be transferable to later use on the cell level. Herewith, we reveal not only the performance limitations but also the effect of catalyst degradation on the most relevant thermodynamic parameters.

The structure is as follows: after presenting the model and its parameterization using experimental CV results, the energy profile alongside the OER reaction coordinate is revealed, and the limiting surface processes on the pristine IrO_2_ material over a wide potential range are identified. Due to the fact that the model approach is not restricted to a certain material state, degradation-related changes of the geometry as well as energy parameters are quantified and the performance losses are traced back to changes of the energies of single reaction steps. As a result, this study provides essential new mechanistic, kinetic, and thermodynamic insights into OER performance at degrading IrO_2_. The versatile model-based methodology is not only restricted to the OER on IrO_2._ We envision it to also be applicable for many other electrocatalysts and electrocatalytic processes and thus as a valuable extension of the available methodologies in electrocatalysis. This study serves as the basis and example for further scientific studies.

## 2 Methods

In this section, the formulation of the OER mechanism, the microkinetic model, and the experimental methods are described. The methodology comprises the following steps: first, the reaction mechanism of the surface processes is identified based on already published studies. Based on the mechanism, rate equations for the processes at the electrode surface are formulated. To reproduce experimental CV data, the reaction rates are determined for a dynamic input signal, a cyclic potential *E*(*t*), by balancing the coverage of all adsorbed surface species. In addition, the resulting time-dependent current density *j*(*t*) is calculated by employing a charge balance. To estimate the unknown kinetic energy parameters and the density of active sites, we use global and local optimization algorithms which identify those values that allow to best reproduce the experimental data. The procedure is used for fresh and degraded catalysts alike.

### 2.1 OER Mechanism

The first step in the methodology comprises the formulation of a detailed OER mechanism on rutile IrO_2_, which provides the basis for the microkinetic model. Over the last decades, multiple OER mechanisms were used to explain the electrocatalytic formation of oxygen from water.^12^ A selection of proposed mechanisms was reported recently for iridium oxide by Naito et al.^33^ Using DFT, a four-step proton-coupled electron-transfer mechanism has been proposed for OER on IrO_2_^11^: on the free Ir coordinatively unsaturated site (CUS) denoted with *, adsorbed intermediates *OH, *O, and *OOH are formed by deprotonation of either water or the adsorbed species itself. Recent DFT studies show process limitations due to oxygen detachment and water adsorption.^13,14^ We found similar limitations with our kinetic model for OER on hydrous IrO_x_.^29^ To take these steps into account, the four-step proton-coupled electron-transfer mechanism of Rossmeisl et al.^11^ is complemented by elementary reaction steps on water sorption and oxygen detachment, which results in the assumption of three additional surface species: *OO, *H_2_O, and *OH_2_O. For latter species, DFT results disagree on whether it is energetically favorable for one of the protons to adsorb on a neighboring *O site^13^ or on the outermost lattice oxygen.^14^ In our mechanism and microkinetic kinetic model, we assume a single site as further discrimination does not affect the kinetics. Explicit consideration of different sites may be conducted in future as refinements to our work using, for example, coupled continuum-kinetic Monte Carlo models.^34^ They would require to take into account further experimental data for discrimination between the mechanisms. The here proposed mechanism is given with eqs 1–7 and consists of two water adsorption steps: eqs 1 and 4, four deprotonation steps: eqs 2, 3, 5, and 6, and the oxygen detachment: eq 7
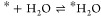
1
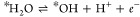
2
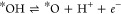
3
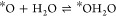
4
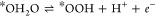
5
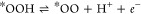
6
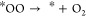
7

### 2.2 Microkinetic Model

In this section, the mathematical model is presented and a detailed explanation of the individual model equations and the underlying assumptions is given. The input function is defined as the electrode potential *E*(*t*) in the form of eq 8
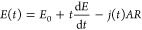
8with a starting potential *E*_0_ and a potential gradient d*E*/d*t* which is held constant in absolute values to simulate CV curves or set to 0 for constant potential simulations. The potential drop in the electrolyte is accounted for with Ohm’s law by an experimentally determined electrolyte resistance *R*, the current density *j*(*t*), and the geometric electrode area *A*. Using formal kinetics and the transition-state theory, the rates of the assumed reactions *i* = {1, 2, 3, ...,7]} are defined in forward *r*_+*i*_ and backward *r*_–*i*_ directions by eqs 9 and 10, respectively
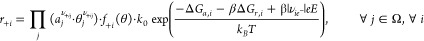
9
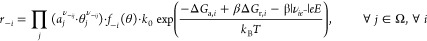
10where *a*_*j*_ is the activity of the electrolyte species *j* ∈ Ω_el_ = {H^+^; H_2_O; O_2_} and θ_*j*_ is the surface coverage of adsorbed species and the free sites * *j* ∈ Ω_sur_ = {*; *H_2_O; *OH; *O; *OH_2_O; *OOH; *OO}, which sum up to unity: ∑θ_*j*_ = 1. The matrix of stochiometric coefficients **ν** = ***ν***_+_ – **ν**_–_ of all species Ω and reactions *r* are given in the Supporting Information, eqs S1 and S2. The reaction free energies Δ*G*_*r*,*i*_ are implemented as the difference in binding energy of the reactant and product species in the forward direction of the assumed reactions. The activation free energy Δ*G*_*a*,*i*_ contains the energy barrier alongside each reaction coordinate in both directions. Further values are the elementary charge *e*, the Boltzmann constant *k*_B_, temperature *T*, the symmetry factor *ß*, and the pre-exponential frequency factor *k*_0_.

To consider the effect of changes in the surface energy state due to areal spacing of the surface sites and the lateral interaction energy of adsorbed species Δ*G*_int,*j*_, the van der Waals isotherm, also known as the Hill-de-Boer isotherm, is adapted to the model in the form of a function *f*(θ), which is described in detail elsewhere.^29,35,36^ It is given for forward *f*_*+i*_(θ) and backward *f*_–*i*_(θ) directions in eqs 11 and 12, respectively.
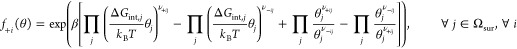
11
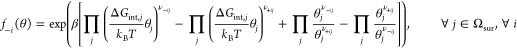
12

The surface coverages of all species are balanced in eq 13, which enables us to solve the set of rate equations dynamically over time.
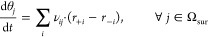
13

In the next step, the dynamic model output, the density of transferred charges over time *j*(*t*), is defined by a charge balance in eq 14 containing the charge accumulation in the specific double layer d*q*/d*t* with its double-layer capacitance *C*_dl_. Further, sinks and sources in charge due to electrochemical reactions at the active surface sites are considered, with ρ being the surface site density with respect to the geometric electrode area *A* and *F* being the Faraday constant.
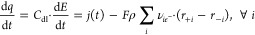
14

### 2.3 Model Parametrization

The final step in the modeling methodology contains the parameterization. Experimental parameters such as electrolyte resistance *R* = 18.2 Ω, electrode area *A* = 0.1963 cm^2^, activity of electrolyte species *a*_H^+^_ = 0.2 and *a*_H_2_O_ = 1, and temperature *T* = 298.15 K can be inserted directly, as well as the stoichiometric parameters, which are deduced from the mechanism. The symmetry factor *ß* = 0.5 and the pre-exponential frequency factor *k*_0_ = 343.2 s^–1^ are defined by the symmetric peak-to-peak position as described in detail in the Supporting Information in Section 1.3 and in Figure S2. The remaining parameters, that is, the energy values, the density of active sites, and the double-layer capacitance, need to be determined from the CV measurements. The process of model-based parameter identification is of major importance to gain a reliable and valid model. Two identification algorithms were combined. In a first step, the dynamic model output of 1 million randomly selected sets of parameters was compared to experimental CV data by evaluating the root-mean-square error (rmse). The 250 sets with the best agreement were further optimized locally by minimizing the rmse with a pattern search algorithm. Details of the overall procedure are given in the Supporting Information in Section 1.3 and in Figure S1. For the parameterization of the processes at the degraded material, the pattern search algorithm is employed and values of the previous modeled state are used as initial parameter values.

To avoid model overfitting by the usage of an unjustified high number of model parameters, we consider four main requirements. First, we strictly use physical parameters which are well established in the recent literature. Second, the mechanism is chosen based on widely accepted insights in the scientific community working on the OER on IrO_2_ and on further materials. Third, we select experimental CV to get the most maximum number of characteristic features to correlate them to kinetic steps. Fourth, we test the model validity by predictions of further experiments such as dynamic CV curves with other scan rates or steady-state polarization curves.

With the described model approach, one is able to reproduce and analyze the ongoing electrocatalytic processes at the electrode surface, which will be discussed in detail in the result section. Prior to this, we will briefly introduce the experimental characterization of the catalytic material.

### 2.4 Experimental Characterization

The IrO_2_ nanoparticles used in this study have been produced by flame spray pyrolysis and have been calcined at 600 °C.^9,10^ In previous publications by Escalera-López et al.^9^ and Czioska et al.,^10^ they have been extensively characterized by physical methods such as transmission electron microscopy, X-ray photoelectron spectroscopy, X-ray diffraction, and operando X-ray absorption spectroscopy as well as by electrochemical methods such as CV and potential steps, whereas the dissolution was analyzed with ICP–MS.

For the experimental electrochemical analysis in this study, we used CV, electrochemical impedance spectroscopy, and chronoamperometry measurements. All electrochemical experiments were conducted with a working electrode from PINE research Instrumentation Inc., which consists of a glassy carbon disc electrode tip fixed in a PEEK shroud with an available circular area of *A* = 0.1963 cm^2^. Back-sided electric connection to a Gamry Reference 600+ potentiostat was ensured via the rotator shaft of a rotating disc electrode setup from PINE research Instrumentation Inc. Deionized water (16 MΩ cm) was used for rinsing the PTFE cell prior to the experiments and as a solvent to prepare the aqueous 0.1 M H_2_SO_4_ electrolyte solution from concentrated sulfuric acid (98%, Carl Roth).

The catalyst ink was prepared as described by Escalera-López et al.^9^ by weighing 2 mg of the IrO_2_ nanoparticles and adding 750 μL of deionized water, 250 μL of isopropanol, and 8.58 μL of Nafion 5% dispersion (D-520, VWR). Further, 1.2 μL of 1 M KOH is added to achieve a pH value of ca. 11, which is reported to homogenize particle distribution on the electrode.^37^ After ultrasonicating for 10 min, 10 μL of the dispersion was dropped onto the glassy carbon electrode, which had been mirror-polished with 0.05 μm alumina suspension prior to drop coating. To gain a uniform film distribution,^38^ the electrode was rotated at 700 rpm for 30 min during drying under atmospheric conditions. The procedure resulted in a catalyst loading of approximately 0.1 mg_cat_ cm^–2^.

For the electrochemical experiments, a Pt wire and a HydroFlex reversible hydrogen electrode (RHE) from Gaskatel GmbH served as counter and reference electrodes in the 250 mL aqueous 0.1 M H_2_SO_4_ electrolyte solution, respectively. Electrochemical analysis of the pristine catalyst material was conducted by, first, potentiostatic impedance spectroscopy at the open-circuit potential with frequencies from *f* = 10^5^ to 10^–1^ Hz and a perturbation amplitude of *E* = 10 mV. Second, three consecutive cyclic voltammograms were recorded at each of the following scan rates: d*E*/d*t* = {200, 100, 50, 25, 200} mV s^–1^ in between potentials of *E* = 0.05 and 1.60 V. All potentials are given with respect to RHE and were *iR*-corrected after the measurements by the electrolyte resistance *R*, which in turn was gained from the impedance spectra at high frequencies at a phase angle of φ = 0°.

Degradation of the catalyst was monitored with the following protocol: first, potentiostatic impedance spectroscopy at the open-circuit potential was conducted as described before to monitor electrolyte resistance. Next, three consecutive cyclic voltammograms with a potential scan rate of d*E*/d*t* = 200 mV s^–1^ in between potentials of *E* = 0.05 and 1.60 V were performed, followed by holding a constant operation potential of 1.2, 1.5, 1.55 V, or 1.6 V for 30 min. The variation in operation potentials allows to study conditions at which no, low, moderate, and high OER activity can be expected, respectively. This step was repeated 30 times, which resulted in 30 sets of CV measurements over an operating time span of roughly 15 h for each operation potential. For better visualization, only cyclic voltammograms after every 150 min and only the last of three CV measurements of the respective set will be shown in the result section. At the end of the degradation test, the impedance measurement was repeated to reveal possible changes in the electrolyte resistance. The electrode was rotated at 2000 rpm during the complete protocol to ensure fast electrolyte transport and avoid the blockage of catalytically active area by evolving oxygen bubbles.

For characterization of the steady state, polarization curves were measured under a constant rotation of 2000 rpm. To guarantee reproducibility, the protocol consists of two subsequent sequences of 17 constant potential steps, where each potential is held for 120 s. The first sequence was conducted by starting at 1.4 V and increasing by 0.025 V up to 1.8 V, and the second sequence was conducted by decreasing the potential after each step by 0.025 V back to 1.4 V. The measurement was repeated three times on freshly prepared electrodes, and the current measured at the end of each potential step was used to calculate the mean value and standard deviation over all measurements at a certain potential.

## 3 Results and Discussion

In this section, we first discuss the experimental CV; then the identified model parameter values are analyzed, and the interplay of surface processes and their impact are revealed under dynamic and steady-state conditions. In the last subsection, this analysis is extended to degraded catalyst states, in which we explain the impact of material degradation on the electrocatalytic performance and parameters.

### 3.1 Experimentally Observed Electrocatalytic Behavior

**Figure 1:**
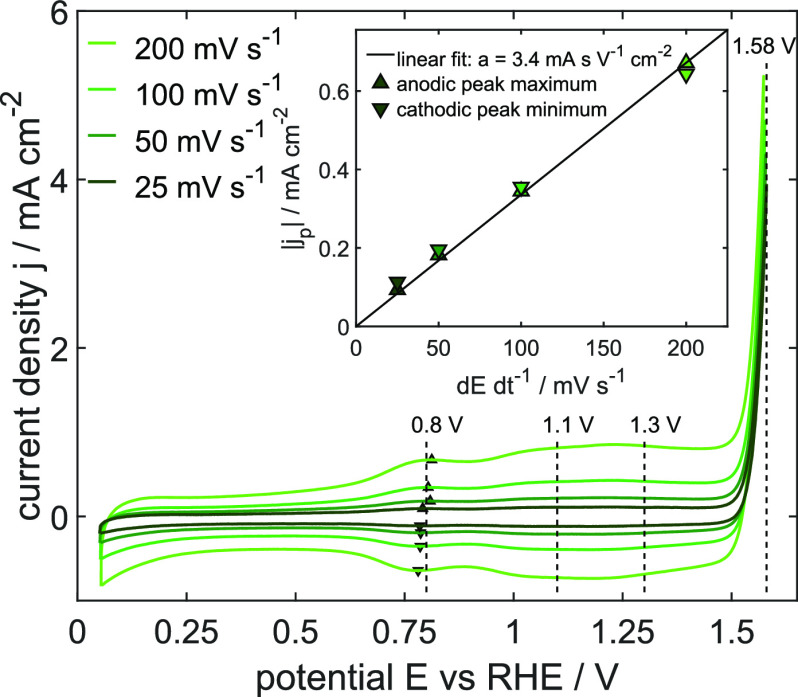
Cyclic voltammograms of pristine rutile IrO_2_ nanoparticles in 0.1 M H_2_SO_4_ at different scan rates. The inset shows the linear current behavior of the absolute peak maximum at around 0.8 V. All potentials are referred to RHE and are *iR*-corrected.

In the following, the experimental CV curves are analyzed to identify features which correspond to electrochemical reactions and analyze the changes in these features which relate to catalyst degradation. Figure 1 shows the third cycle of CV curves at various potential scan rates. The *iR*-corrected OER overpotential is quantified to 350 mV at the lowest potential rate of 25 mV s^–1^ and at a current density of 4 mA cm^–2^ or roughly 40 mA mg_cat_^–1^, which is similar to previously reported data with minor deviations due to the particle size and geometric area.^5,39,40^ Currents in 0.25 V < *E* < 1.5 V versus RHE depend linearly on the applied potential scan rate, which is exemplarily shown for the maximum peak position at 0.8 V in the inset of Figure 1, and are, therefore, attributed to pseudocapacitive processes. Constant current contributions at 0.25 V < *E* < 0.5 V occur due to charging of the double layer. Above 0.5 V, three partially overlapping redox transitions are visible at roughly 0.8, 1.1, and 1.3 V, which are reported in the literature as subsequent deprotonation steps oxidizing the Ir CUS and the adsorbed oxygen.^19,41−43^ The transition at approximately 0.8 V is correlated to the first deprotonation step *H_2_O ⇌ *OH + H^+^ + e^–^. This is in good agreement with DFT calculations of this reaction on (110) IrO_2_, which gave reaction free-energy values of Δ*G*_r_ = 0.67 to 0.88 eV, depending on the employed revised Perdew–Burke–Ernzerhof functionals accounting for van der Waals interactions and assuming the presence of explicit water.^15^ The broad response in current in 0.9 V < *E* < 1.5 V covers approximately double the amount of transferred charges of the previously discussed transition. It is thus attributed to two deprotonation steps *OH ⇌ *O + H^+^ + e^–^ and *OH_2_O ⇌ *OOH + H^+^ + e^–^ with higher reaction free-energy values, ranging from Δ*G*_r_ = 1.21 to 1.56 eV and from Δ*G*_r_ = 1.26 to 1.68 eV, respectively.^15^ The exponential current increase at potentials *E* > 1.5 V implies that all species in the circular mechanism are rapidly reacting in the forward direction, driving the formation of molecular oxygen. In conclusion, the CV curves show four different electrochemically limited current response features and provide a substantial data set to identify the model parameters. This will allow us to differentiate further processes by the model-based approach, which are not easily accessible with experiments, and to analyze their interactions and performance limitations of the pristine IrO_2_.

**Figure 2:**
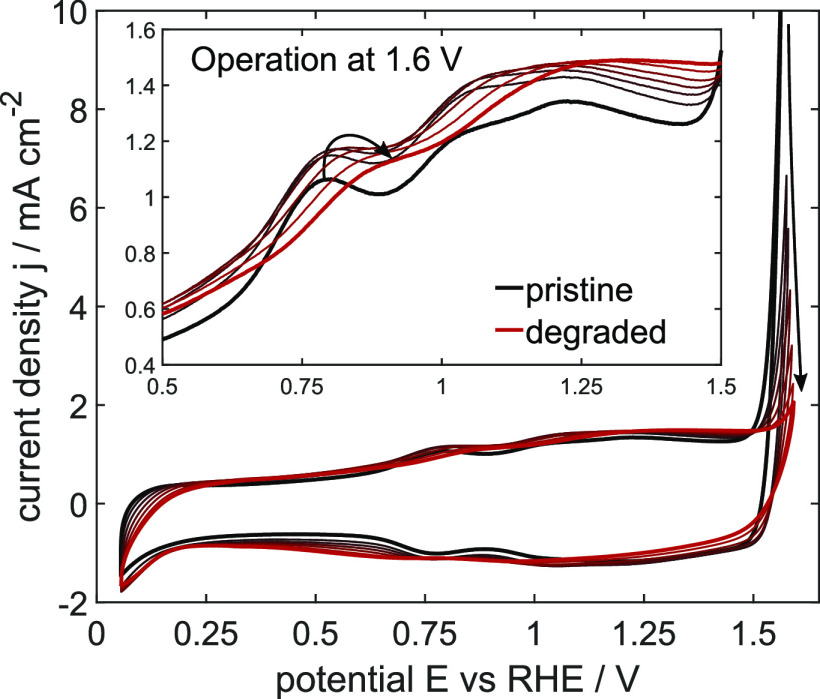
Changes in experimental CV curves with a scan rate of 200 mV s^–1^ during 15 h of OER operation at a constant potential of 1.6 V versus RHE. The third CV curve recorded after every 150 min is displayed, with colors changing from pristine (black) to the degraded state (red); changes are indicated by arrows. The inset magnifies the shift in anodic redox transitions.

One of the major concerns regarding the actual performance of a catalyst material is the long-term stability. Therefore, the electrochemical long-term behavior of the IrO_2_ nanoparticles was monitored by (i) applying a constant potential at which the OER occurs for 15 h in total and (ii) measuring during these 15 h cyclic voltammograms every 30 min in order to gain a dynamic current response of the steadily degrading system. The resulting CV curves in Figure 2 show a strong decrease in the OER current density at the maximum applied potential of 1.6 V from 10.4 to 2.1 mA cm^–2^_._ This follows other literature reports stating that the catalytic system undergoes strong degradation in acidic media.^44^ Interestingly, at potentials below OER, a slight absolute increase in the redox transition current is observed, alongside a shift of the anodic deprotonation peak positions toward higher potentials (inset of Figure 2) and of the cathodic peak positions toward lower potentials. Both effects, the decrease of OER current and the gradual change in the redox transitions, are strongly dependent on the applied operation potential: experimental results at lower operation potentials reveal significantly smaller changes and thus lower degradation, as shown in the Supporting Information in Figure S3. This change in performance cannot be attributed to electrolyte concentration changes, as discussed in the following. The electrolyte resistance before and after all long-term measurements remained almost constant, for example, *R* = 18.2 to 18.4 Ω for 1.6 V. Therefore, a significant change in concentration of protonic charge carriers and, thus, pH value is disproved. Further, the local oxygen concentration gradient is held constant due to a high electrode rotation speed of 2000 rpm. One might argue that the formation of oxygen micro-bubbles leads to a decrease in catalytic currents, as recently reported.^8^ However, in our experiments, it is clearly shown that the absolute current of the redox transitions does not decrease and, thus, possibly produced bubbles at high potentials are either reduced completely while applying reductive potentials prior to the third of the consecutive cyclic scans or transported away from the electrode by the fast electrode rotation. Also, only a slight recovery of the activity is observable in the Supporting Information in Figure S4 while applying a moderate constant potential of 1.2 V for 5 h after the OER operation for 15 h. Anyhow, no major blockage of active surface sites is detectable in the CV curves. In summary, this change in dynamic current behavior due to long-term operation is attributed exclusively due to a progressive degradation of the catalytic material itself.

In the following section, two remaining aspects will be tackled by the model-based analysis: first, which microkinetic processes are affected by this material degradation, and second, how to quantify the change leading to this loss in activity with physically meaningful values. Beforehand, we will discuss the parameters and the simulation output of the pristine material, which reveal the performance-limiting steps.

### 3.2 Energies and Further Model Parameters

**Figure 3:**
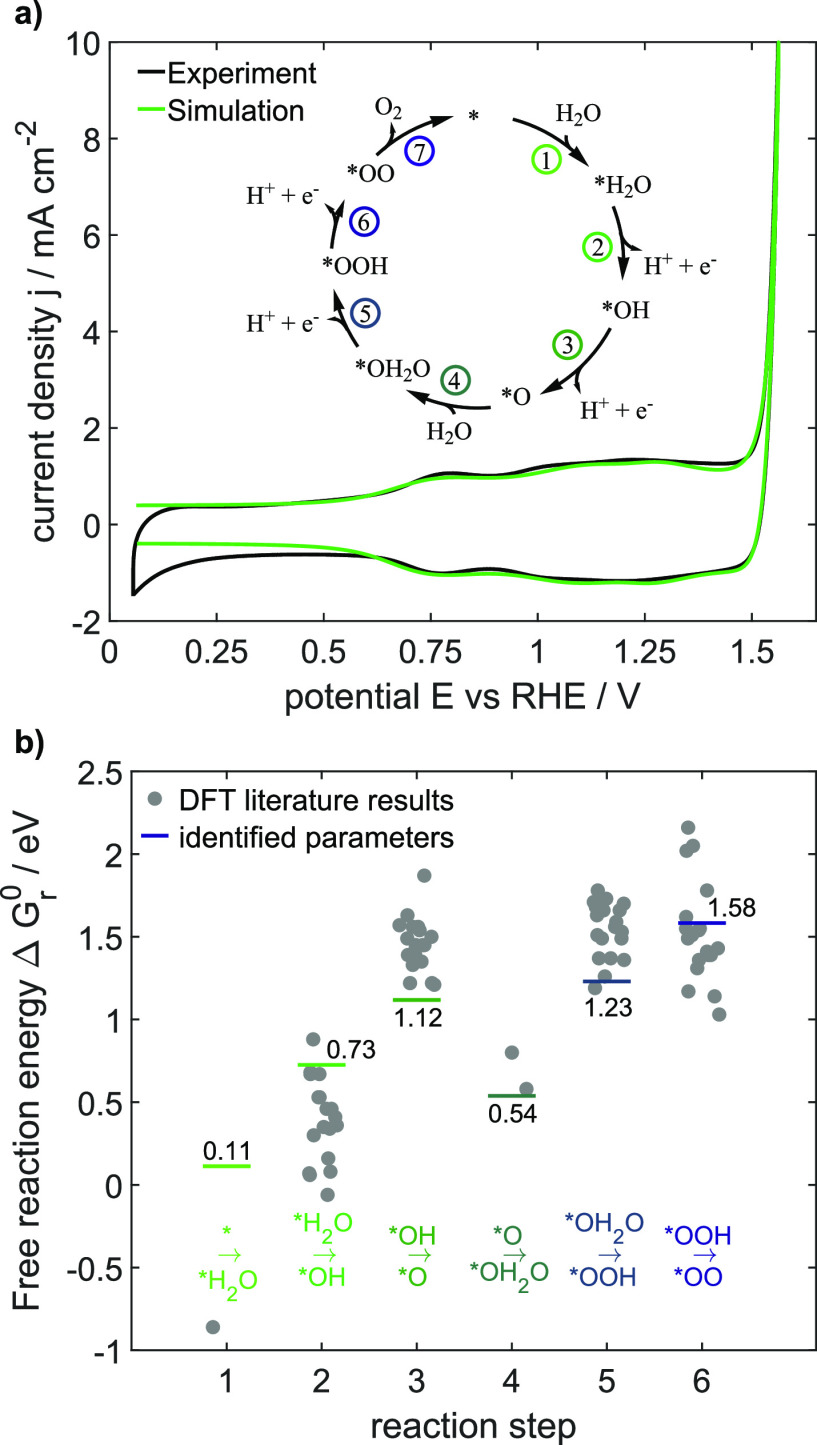
(a) Comparison of the experimental and simulated CV curves for a scan rate of 200 mV s^–1^ at the pristine state with the best set of parameters. (b) Identified reaction free-energy parameters for steps 1–6 at electrochemical standard conditions in comparison to DFT-calculated values reported in the literature.^13−15^ Adsorbed reactants are displayed for each of the reaction steps given in eqs 1−7.

With the parameter estimation procedure described in the method section, a set of parameters was elaborated, which allows to reproduce the experimental CV data with extremely low deviation (an rmse value of 0.066 mA cm^–2^) as seen in Figure 3a. All major features, the redox transitions, and the exponential increase of the OER are reproduced. The soundness of the model and its parameterization are further confirmed as the simulations with the same parameter set can also reproduce experimental cyclic voltammograms at different scan rates: the scan-rate dependence of the cyclic voltammograms and the features matches nicely, as shown in the Supporting Information in Figure S5. This is a further clear indication that the model is not overfitted. In addition, profile rmse analysis was conducted on the reaction free-energy parameters. The results given in the Supporting Information in Figure S6 confirm the high parameter identifiability. The resulting energy parameter values for the pristine catalytic system are listed in Table 1 and will be discussed in the following paragraphs to give a sound analysis of the model-based findings.

**Table 1:** Values for the Reaction Free Energy, the Activation Free Energy, and the Reactant Species Interaction Energy of Reaction Steps (1)–(7) Identified by Reproducing CV with the Microkinetic Model^a^

step	Δ*G*_r_^0^ / eV	Δ*G*_a_ / eV	Δ*G*_int_ / eV
(1)	0.11	0.15	0.18
(2)	0.73	0.00	0.10
(3)	1.12	0.00	0.11
(4)	0.54	0.00	0.13
(5)	1.23	0.00	0.00
(6)	1.58	0.04	0.03
(7)	–0.39	0.43	0.02

a Further identified parameters are the double-layer capacitance of *C*_dl_ = 19.4 F m^–2^ and the density of active sites of ρ = 1.40·10^–4^ mol m^–2^.

Figure 3b allows a comparison of the reaction free energies at electrochemical standard conditions to reported values obtained by DFT. The high fluctuations in the DFT-based values are due to different revised Perdew–Burke–Ernzerhof functionals. For all electrochemical processes, that is, reaction steps and eqs 2, 3, 5, and 6, the energies estimated by our kinetic model are in good accordance with the DFT studies.^14,15^ Also, the reaction energy of the second water adsorption step, eq 4, matches with the range of reported data. Only the first water adsorption step, eq 1, differs roughly 1 eV from the single available DFT-based literature value.^13^ To the best of our knowledge, no further values are reported to compare with, probably because the intermediate species *H_2_O was often neglected in DFT studies as it is not electrochemically limiting. As the DFT values for the other steps showed strong deviations and thus reliance on a single DFT-value is not recommended, we evaluated a wide range of −3 eV up to 1 eV for this parameter, in which a value of 0.11 eV was identified to describe the dynamic behavior best. We conclude that our methodology enabled indeed to identify the free reaction energies of the mechanism, while nicely describing the dynamic behavior.

Activation free energies describe the energy barriers of transition states along a reaction coordinate. For all electrochemical deprotonation processes, we identified low values of 0.04 and 0 eV as shown in Table 1 and, thus, no or marginal barriers hamper the protons to desorb. This is in good agreement with a barrier of <0.05 eV reported in a recent DFT study.^16^ Water adsorption steps are also found to face marginal activation energies of Δ*G*_a,1_ = 0.15 eV or Δ*G*_a,4_ = 0 eV. In contrast, a high activation energy Δ*G*_a,7_ = 0.43 eV is identified for the oxygen detachment process (*OO → * + O_2_), meaning that the microkinetic reaction rate is restrained by an additional energy barrier alongside the reaction coordinate, which is in accordance to a recently reported DFT study.^14^

The interaction energies of adsorbed species Δ*G*_int_ are also identified with the model-based approach and are given in Table 1. Derived by Frumkin^45^ and de Boer,^35^ the interaction energy in eqs 11 and 12 affects the adsorption process with increasing coverage of the adsorbed species. A physically meaningful ascription is given by two independent interpretations: (i) de Boer stated that the energy covers lateral molecular interactions, nowadays known as van der Waals forces.^35^ This explanation does hold for the studied catalytic system since permanent charges and dipoles are involved in the adsorption process. (ii) Temkin, in contrast, accounts for a non-uniform catalytic surface.^46^ As for IrO_2_ nanoparticles, different surface orientations, (110), (101), and (100), are reported by analyzing the Wulff construction;^47,48^ also, the explanation by Temkin is applicable for this material. In a recent DFT study by Rao et al. on electrocatalytically highly related RuO_2_, free energies of adsorbed species occurring in the OER mechanism were found to differ due to the different assumed facet with values ranging from 0.02 eV up to 0.33 eV.^49^ In conclusion, both reported interpretations hold for the studied catalytic system and might influence the adsorption process. The interaction energy values were identified for the multiple species as given in Table 1 and range from 0 eV for *OH_2_O to 0.18 eV for the free active site *. This is in the range of values reported for the adsorption of different alcohols.^50,51^ From a microkinetic point of view, a higher interaction energy of a reactant species increases the reaction rate initially due to high reactant coverage but lowers the rate by the ongoing production of the product species. This behavior leads to the broadened current response of the electrochemical reactions shown exemplarily for a proton-coupled electron-transfer step in Figure S7. Although the microkinetic analysis is able to quantitatively identify the interaction energies very precisely, future research may focus on this parameter to elucidate its origin and to prospectively resolve whether the behavior is caused by different surface facets or the impact of van der Waals interactions of neighboring species.

The estimated specific double-layer capacitance is *C*_dl_ = 19.4 F m^–2^ with respect to the geometrical electrode area. It is in good agreement to previously reported data.^52^ The density of active sites is quantified to ρ = 1.40 × 10^–4^ mol m^–2^, which corresponds to 84.2 sites per nm^–2^ with respect to the geometrical electrode area. Assuming nominal particles with an iridium to oxygen ratio of 1:2, a percentage of 3.1% of all iridium atoms contained in the particles serves as active sites for electrocatalysis. To evaluate the electroactive surface area (ECSA), a method reported by the group of Bandarenka^53^ was applied (see details in the Supporting Information in Section 2.5 and Figure S8) using their reference value for the specific adsorption capacitance of IrO_*x*_. The ECSA of the present catalytic system is 2.7 ± 0.6 cm^2^ and, consequently, 13.7 ± 2.8 times larger than the geometrical electrode surface. By combining this value with the model-based identified density of active site, the actual density value normalized to the ECSA of ρ_ECSA_ = ρ·*A*·ECSA^–1^ = 6.2 ± 1.3 nm^–2^ is received. This value can now be directly compared to reported literature values. It is in good agreement with the reported iridium CUS density in rutile IrO_2_ (110) and (100) facets of 5 and 7 nm^–2^, respectively.^54^ The large uncertainty arises due to the quantification of the specific adsorption capacitance from the impedance spectra.

The above given in-depth analysis and literature comparison proves that the model-based parameter identification process applied on CV curves provides reliably estimated values of the thermodynamic energies, the double-layer capacitance, and the density of active sites. The physically meaningful parameters are in overall good accordance with reported data and describe independently different aspects of the catalytic system. Thus, it is conclusively shown that the present model is not overfitted. With this fully parameterized physicochemical model, we will analyze in the following the interplay of reactions and surface species and the resulting impact on the performance and its kinetic limitations.

### 3.3 Interplay of Surface Processes and Their Kinetic Impact

**Figure 4:**
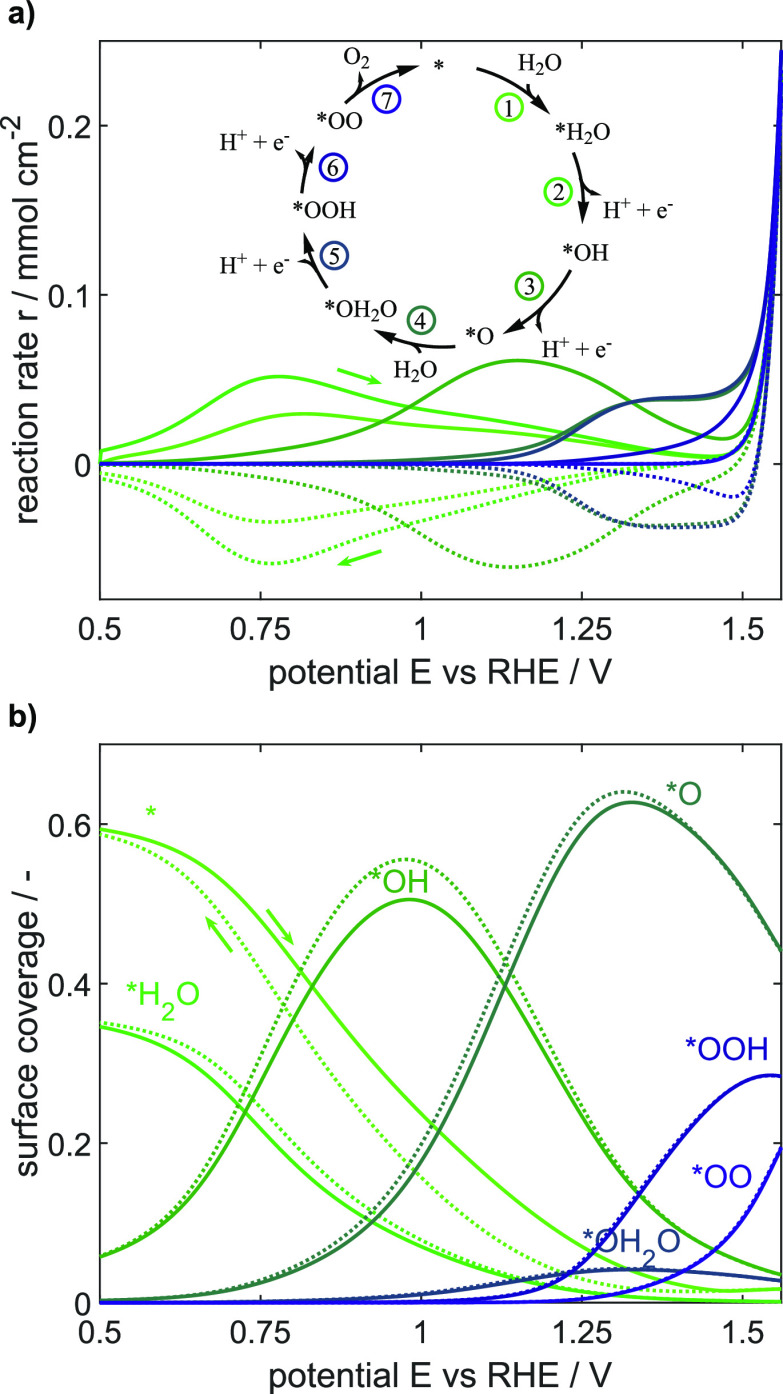
Simulated (a) effective reaction rates according to the color-coded mechanism in the inset and (b) surface coverages of adsorbed species during a cyclic voltammogram with a scan rate of 200 mV s^–1^. Solid and dashed lines indicate forward and backward potential cycling directions, respectively, as indicated by the arrows.

To gain an understanding about the relationship between electrochemical behavior, performance of the catalyst, and the microkinetic processes, a model-based analysis is conducted. The advantage of a parameterized microkinetic model is the possibility to analyze with it the behavior and interactions of elementary reaction steps as well as single limitations that affect the overall electrochemical behavior and performance at a given potential. For this purpose, CV simulations with a scan rate of 200 mV s^–1^ are analyzed to provide insights into the reversible and potential-dependent changes in reaction rates and the surface coverage of adsorbed species. The evolution of these variables over the full third cycle is shown in Figure 4.

A high reversibility of all reactions can be concluded from the similar coverage curves for forward and backward scans and the corresponding mirrored reaction rates. Furthermore, it is clearly observable that at different potentials, individual reactions kinetically limit the electrocatalytic conversion. At low potentials up to 0.9 V, water adsorption (* + H_2_O → *H_2_O) and the following deprotonation step (*H_2_O → *OH + H^+^ + e^–^) are predominant, resulting in an increase in adsorbed *OH. In the potential range from 0.9 V up to 1.3 V, the second deprotonation process (*OH → *O + H^+^ + e^–^) sets in and becomes predominant, resulting in a high amount of *O covering the surface’s active sites with a share of up to 60% at ∼1.3 V. The stepwise oxidation is accompanied with an increase in the mean oxidation state of Ir CUS by roughly 1.6. We confirm this trend by performing X-ray adsorption measurements, which result in a corresponding shift of the absorption edge in operando X-ray absorption spectra, see the Supporting Information in Figure S9. The connecting point between microkinetic modeling of IrO_2_ and operando spectroscopy is a helpful link to correlate insights and study further catalytic materials. With the further increase of the potential above 1.3 V, the amount of *O slightly decreases to 44% and the amount of *OOH increases. As two deprotonation processes, that is, production of *O and of *OOH, occur predominantly in the potential range from 0.9 V up to 1.5 V, the experimentally observed broadened current feature comprises the transferred charges of both processes. Above 1.5 V, the further reactions including oxygen release set in, which finally leads to an exponential rise of oxygen evolution and thus the overall OER turnover frequency. The fact that *O only slowly decreases and very few *OH_2_O can be observed suggests that water adsorption partially limits the OER. This outcome is expectable as in contrast to the electrochemical steps, which accelerate with potential, chemical rates are not directly dependent on the applied potential. In addition, the fourth deprotonation step (*OOH → *OO + H^+^ + e^–^) is observed to limit the overall OER electrochemically as its rate increases rather only slightly at a high potential of 1.5 V and, hence, it contributes significantly to the overpotential of the OER. The limitation is also manifested in the high coverage share of 28% of adsorbed *OOH at 1.56 V. At the highest simulated potential of 1.56 V, *OO species accumulate and cover a share of 20% of the surface. This indicates a third limitation in the oxygen detachment step (*OO → * + O_2_). It is worth mentioning that this step is not explicitly influenced by the applied potential as no electrons are transferred; however, it is indirectly impacted as its rate depends on the coverage of the surface with the reactant species *OO, which gets significantly increased with a higher potential as shown in Figure 4b.

**Figure 5:**
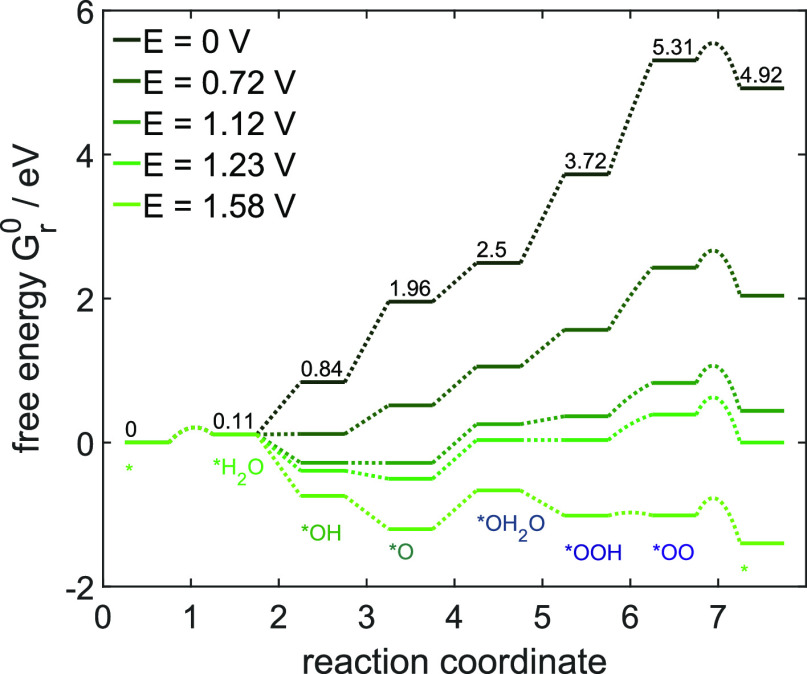
Energy diagram for the oxygen evolution microkinetics for potentials between 0 and 1.58 V vs. RHE. Displayed are the cumulated reaction energies (plateaus) and activation energies (dashed lines) of the single steps (eqs 1–7). The numbers given at *E* = 0 V are the free-energy values at electrochemical standard conditions of *a* = 1 and *T* = 25 °C.

An intuitive way to visualize the limitations by electrochemical and chemical steps as a function of potential is shown in the energy diagram in Figure 5. Such representation allows to easily study the changes in energy levels with respect to potential. Figure 5 shows that the low activation energies of deprotonation steps facilitate the highly reversible microkinetics. The only notable activation barrier is found to be present for the oxygen detachment step, given in eq 7. It leads to a limitation in the OER. At high potentials of 1.58 V, besides this step, only the reaction energies of both water sorption steps pose an additional notable energy barrier. All three limitations can be kinetically overcome by increasing the amount of the respective reactant species. However, this is at the cost of the subsequent reactions: for example, the third deprotonation step (*OH_2_O → *OOH + H^+^ + e^–^) proceeds at a potential roughly 0.5 V higher compared to the second deprotonation step (*OH → *O + H^+^ + e^–^) shown in Figure 4a, although both reaction energies differ only by about 0.11 eV. Here, the kinetic analysis provides further input to describe the interactions. The scarce availability of *OH_2_O due to the sluggish water adsorption process consequently increases the potential at which the subsequent electrochemically driven reaction kicks in. This is particularly relevant at the highest potential of 1.58 V: here, all electrochemical reactions are thermodynamically favorable, but the high amount of the reactant species *O and *OO, which are required for the two chemical steps of water adsorption and oxygen detachment, respectively, indicates that especially these steps limit the overall OER cycle. Since no electrons are transferred in both steps, they are not explicitly accelerated by higher applied potentials. The reader should bear in mind that this is a dynamic scan. Whether the limitations are similar during steady-state operation will be analyzed in the following section.

### 3.4 Polarization Behavior

**Figure 6:**
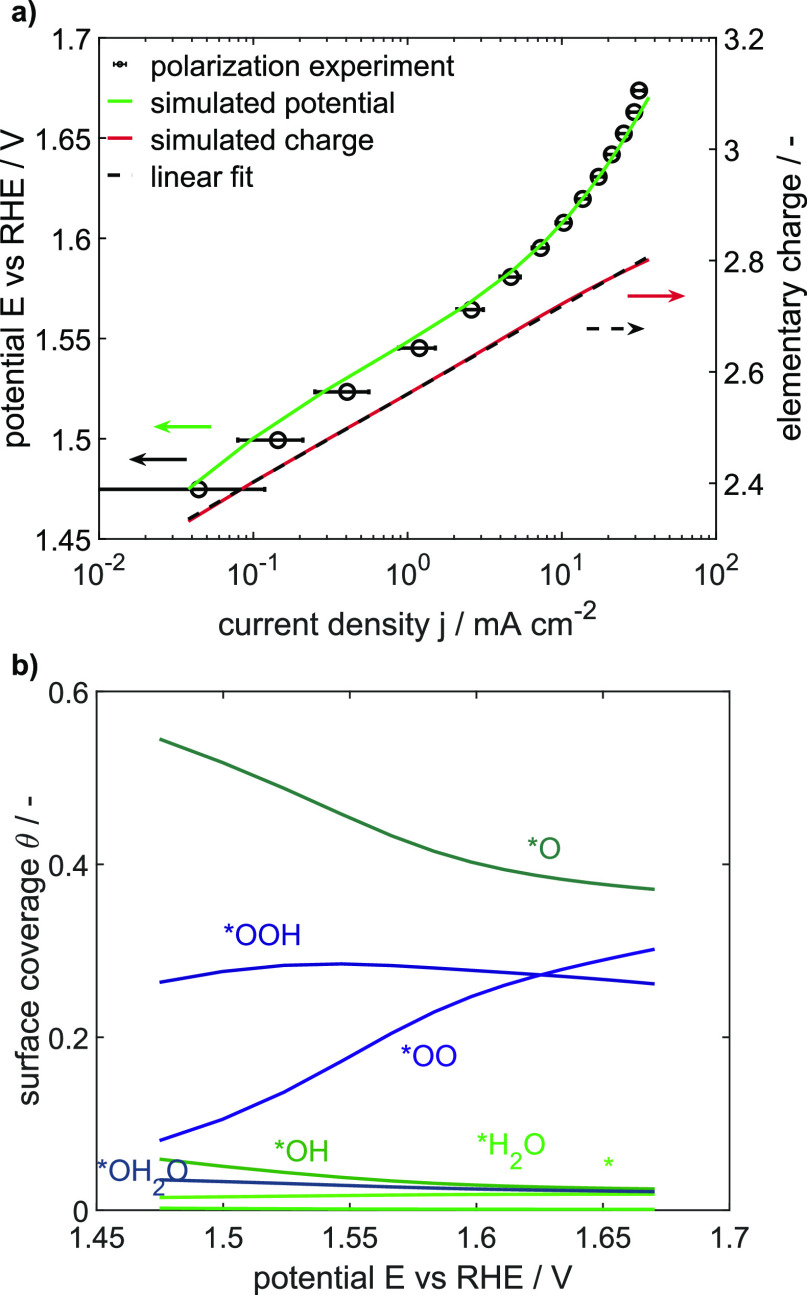
a) Experimental and simulated polarization curves for technically relevant potentials and the simulated change in elementary charge per active site. Experiments were repeated three times. (b) Corresponding surface coverages of the steady-state simulation.

Cyclic voltammograms are inherently dynamic and do not show steady-state behavior and limitations as they would occur during practical operation of PEMWE. Here instead, steady-state measurements such as a polarization curve are of more help. We therefore analyze the CV-parameterized model for its steady-state performance and check whether it can reproduce experimental behavior and aim to analyze the underlying loss processes. This is accomplished by comparing experimental polarization results to simulated ones, as shown in the Tafel plot in Figure 6a. The original energy parameter set from the CV simulations was taken, and a good match was achieved between the experiment and simulation. This proves that the model, which was parameterized with CV curves up to 1.58 V, is not only able to reproduce steady-state behavior in the same range but also able to predict even the steady-state currents at higher OER potentials such as 1.67 V. This positive outcome confirms the validity of the presented OER model and its parameters. It should be further noted that – as to be expected for such a complex process – the simple approach of mapping the kinetics to a single Tafel slope is not feasible: both curves show a slightly curved profile with a continuously changing slope, which is especially visible for the simulation. Thus, there is not a single Tafel slope across the OER operating window, neither experimentally nor in simulation. This corresponds to recent analysis of published Tafel slopes for CO_2_ reduction, which showed an extremely broad distribution between ca. 30 and 200 mV/dec.^55^ It is thus highly recommended to conduct model-assisted microkinetic analysis, as given in this work, to understand such limitations.

The corresponding coverages of surface sites during the OER are also given in Figure 6b and show interesting additional insights: the surface is mostly covered by species *O, *OOH, and *OO, which corresponds to slow water adsorption on *O, slow deprotonation of *OOH, and slow *O_2_ desorption.

While most of the species remain at a rather constant amount, the share of adsorbed oxygen *O continues to decrease with a potential down to 37% and; conversely, the share of the *OO species increases up to 30%. As a consequence, this replacement leads to a change in accumulated charge in the surface species. By calculating the number of accumulated elementary charges per active site, we find a logarithmic increase in the number of elementary charges per active site with current density, increasing from 2.3*e* at 1.47 V to 2.8*e* at 1.67 V, as displayed in Figure 6a (details see Section 2.7 and Figure S10 in the Supporting Information). This logarithmic trend was recently reported as a common relation of the OER^19^ and further confirms the validity of our microkinetic model. This model-based analysis shows clearly that not only the potential-driven reaction (*OOH → *OO + H^+^ + e^–^) is limiting but also the two chemical steps, the water adsorption on *O and O_2_ detachment, are limiting. We therefore propose that an increase in OER activity and performance of the Ir oxide can be reached by improving the ability to adsorb water and detach *OO species efficiently. We conclude that the microkinetic model is a powerful and versatile tool for in-depth analysis of OER behavior of Ir oxide. This brings us to the last part of this study, where we use the model to answer the question on how the kinetic behavior changes during catalyst degradation.

### 3.5 Kinetic Changes Due to Degradation

Understanding the process of degradation of a catalyst and its performance opens the opportunity for knowledge-driven improvement of its long-term stability. A loss in activity may be associated with a change in the mechanism,^56^ which would require to adapt the model equations. Alternatively, it may be related to a loss in active sites or in their ability to catalyze the reaction steps efficiently. The latter would be reflected by a change in the model parameters for the kinetics (e.g., free energies) or of geometric specifications (e.g., density of active sites). Finally, external conditions, for example, electrolyte-related values such as proton concentration and conductivity, might have changed, whereas the model equations and parameters remain valid. To evaluate if the experimentally observed degradation can be attributed to a change in mechanism, kinetics, or electrolyte, we checked if adjusting certain sets of parameters of the pristine system allows to fully reproduce the experimental CV data of the degrading system. If reproduction is possible, the mechanism is valid, and changes in performance can be attributed to kinetics, geometry, or electrolyte depending on the affected parameters. For the analysis, different combinations of parameters were changed (see the detailed discussion in Section 2.8 and Figure S11 in the Supporting Information). Adjusting the density of active sites alone or in combination with the resistance or electrolyte concentration did not allow to explain the experimentally observed degradation behavior. Nevertheless, in all the following analyses, the density of active sites is also adjusted to account for the known and likely degradation effects of material dissolution,^9^ particle detachment,^57^ particle cracking, and loss of binder material.^58^ By analyzing both cases of changing the electrolyte resistance and double-layer capacitance, the simulated behaviors do not match with experimental observation. Thus, degradation is not related to a change in the electrolyte properties.

**Figure 7:**
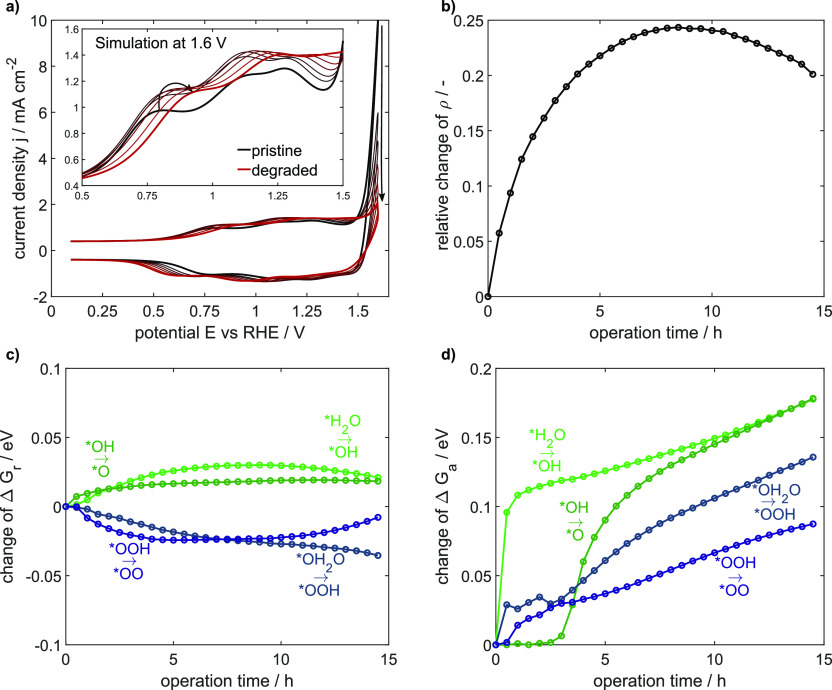
Simulation of the effect of degradation of Ir oxide catalyst during 15 h operation at 1.6 V. (a) Changes in the cyclic voltammogram from pristine (black) to degraded (red) correspond to the states after operation for each 150 min, (b) relative change of site density, and changes in (c) free reaction energies and (d) activation energies.

We found the activation free energy to be the best descriptor for the degradation process, whereas changing only the reaction and the interaction free energies showed significantly higher deviations from experiments. Reducing the full set of energy parameters to only deprotonation-related parameters was found to hardly increase the errors, which indicates that deprotonation processes are significantly impacted by catalyst degradation. On this knowledge base, a combination of parameters, including the density of active sites and activation and reaction free energies of the deprotonation processes, was evaluated. The reproducibility was drastically improved and led to excellent reproduction of the experimental CV. The simulated cyclic voltammograms in Figure 7a present all experimentally observed degradation features: the drastic decrease in OER current density, the slight absolute increase in redox transition currents, and the shift of anodic peak position toward higher and cathodic peak position toward lower potentials. The parameters which changed and are consequently responsible for replicating the degrading CV performance can be individually assessed. The density of active sites increases up to 20% after 8 h of operation, as shown in Figure 7b. The significant increase is most likely attributed to a loss of binder material, which leads to faster exposure of active material than dissolution^9^ of active material. A decrease of active material provoked by dissolution is, thus, excluded to be responsible for the observed performance decrease of the catalyst. The reaction free energies of all four deprotonation steps reveal only minor changes below 0.04 eV of the value for the pristine material in Figure 7c, which corresponds to <2.8% relative changes. The most significant changes are exhibited in Figure 7d with the changes in activation free energies of all four deprotonation steps. There is a clear correlation between time of degradation and activation free energy. After a fast increase during the first hours, a linear increase in activation energies is observed for all four activation energy parameters; slopes are similar for all four steps. This observation of changes in electrocatalytic material properties corresponds well to an experimentally reported partially reversible formation of oxygen vacancies during degradation, which was reported to occur on rutile IrO_2_ nanoparticles under constant potential operation at 1.6 V.^10^ Two likely pathways were proposed. One possibility would be the formation of a lattice oxygen vacancy by saturating a vacant CUS site * to form *O. In the second proposed pathway, molecular O_2_ is formed by combining an oxygen from the already *O-occupied site with a lattice oxygen atom. As in the present study a high coverage of *O is observed at such high potentials, the observed degradation is attributed to the formation of oxygen vacancies by combining oxygen from the *O-covered site with a lattice oxygen atom to form molecular O_2_. As the formation rate of the oxygen vacancies is rather slow compared to the overall OER rate, the results at high potential are insufficient to prove that formation via the free surface species * is negligible. However, as we do not see a significant amount of degradation at a low potential, where free sites * are prevalent, and as degradation monotonically correlates to potential, the degradation is most likely related to the *O-covered sites, which are prevalent at high potentials. The amount of one of the species being present at the surface does not correlate with the formation of oxygen vacancies, but higher potentials do. The oxygen vacancy formation process and thus the degradation are identified as potential-driven.

Major conclusions can be drawn from the above-given analysis. First, the assumed mechanism is able to describe the electrocatalytic behavior not only on the pristine IrO_2_ catalyst but also on all transient states during the long-term aging test. Furthermore, the observed loss in electrocatalytic activity is correlated with a catalyst-related degradation process. This was correlated here to the significant increase in the density of active sites and the activation free energy of deprotonation steps. After several hours of degradation, the change in activation free energy of the elementary deprotonation steps is identified to correlate linearly with time and with the formation of oxygen vacancies^10^ during the degradation process.

## 4 Conclusions

A microkinetic model of the oxygen evolution reaction on IrO_2_ nanoparticles was presented, which elucidates performance limitations by single surface processes and the impact of catalyst degradation on surface processes occurring during OER. In contrast to state-of-the-art approaches, a comprehensive description of the kinetics, thermodynamics, and their changes due to degradation was identified by the use of experimental data. Electrocatalyst-related parameters, such as thermodynamic energies and the density of active sites, were analyzed, and an in-depth understanding on the dynamic formation of surface species was given.

The identified microkinetic model was shown to be highly robust as it reproduces experimental cyclic voltammograms at various potential scan rates and polarization curves and shows similar trends to X-ray spectroscopic methods. Moreover, the identified set of free-energy values is predominantly in the range of reported values by DFT studies. In contrast to DFT calculations, the parameters were determined experimentally. The model yielded a deep insight into not only thermodynamics but also kinetic limitations. In contrast to kinetic modeling methods relying on steady-state or quasi-equilibrium assumptions, the presented dynamic model enabled to resolve the microkinetic quantities of individual elementary processes. Furthermore, the method was shown to be highly effective to study in depth the decrease of catalyst performance by reproducing experimental degradation. It is thus a highly attractive, complementary method for kinetic and degradation analysis.

Analysis of the simulated reaction rates and surface coverages of adsorbed species indicate three main limitations during the OER: (i) slow water adsorption (*O + H_2_O → *OH_2_O) leads to an accumulation of *O species. (ii) The third deprotonation step (*OOH → *OO + H^+^ + e^–^) is identified as the potential determining step due to the high reaction free energy. (iii) A notable activation energy barrier limits the oxygen detachment (*OO → * + O_2_). Regarding the search for a catalyst with better performance, we suggest to focus on active sites that do not only catalyze the electrochemical deprotonation but that also facilitate the water adsorption and oxygen detachment steps.

Further, analysis of degradation-related changes in CV revealed a catalyst-related loss in activity. The assumed reaction mechanism can also reproduce degraded catalyst behavior and, thus, remains valid for the degraded state as well. The identified change in the parameters demonstrates that the degradation is correlated to a nonlinear increase and a subsequent slower linear increase in the activation free energy of the deprotonation steps. In the study, the main reason for the loss in activity is identified as a material-related change, which is correlated to the formation of oxygen vacancies on *O sites.

Future efforts to develop stable electrocatalytic materials may focus on understanding their degradation process and elaborate strategies to reduce its impact. The present study provides insights even into the thermodynamics and kinetics on a long timescale. It demonstrates that microkinetic modeling is a viable method to understand electrocatalytic surface processes even for degrading material states. The methodology is not limited to OER on rutile IrO_2_; preliminary studies indicate its applicability to other OER catalysts such as RuO_2_ and mixtures of both as well. However, applicability does not stop there; we envision its application to many more electrocatalytic systems that can be characterized well by CV. This study thus also serves as an example and as a physical basis for a wide range of electrocatalytic and kinetic studies.

## Supporting Information Available

Computational details, electrochemical degradation results, CV simulations with different scan rates, profile root mean square error analysis, model parameter variation, quantification of electroactive surface area, oxidation state validation, logarithmic charge–current relationship, and model parameter optimization of the degrading system (PDF)
