# Supplementary material for: Microkinetic Analysis of the Oxygen Evolution Performance at Different Stages of Iridium Oxide Degradation
Source: J Am Chem Soc. 2022 Jul 18;144(29):13205–17. doi: 10.1021/jacs.2c03561 (PMC9335572; doi:10.1021/jacs.2c03561)
Supplement: Supplementary file 1 — ja2c03561_si_001.pdf [file ja2c03561_si_001.pdf]

Supporting Information

A Microkinetic Analysis of the Oxygen Evolution Performance at Different Stages of Iridium Oxide Degradation

Janis Geppert[a], Philipp Röse[a], Steffen Czioska[b], Daniel Escalera-López[c], Alexey Boubnov[b,d], Erisa Saraçi[b,d], Serhiy Cherevko[c], Jan-Dierk Grunwaldt[b,d], Ulrike Krewer[a,]*

[a]Institute of Applied Materials-Electrochemical Technologies (IAM-ET), Karlsruhe Institute of Technology, Adenauerring 20b, 76131 Karlsruhe, Germany

[b]Institute for Chemical Technology and Polymer Chemistry (ITCP), Karlsruhe Institute of Technology, Engesserstr. 20, 76131 Karlsruhe, Germany

[c]Helmholtz-Institute Erlangen-Nürnberg for Renewable Energy (IEK-11), Forschungszentrum Jülich GmbH, Cauerstr. 1, 91058 Erlangen, Germany

[d]Institute of Catalysis Reasearch and Technology (IKFT), Karlsruhe Institute of Technology, Hermann-von-Helmholtz-Platz 1, 76344 Eggenstein-Leopoldshafen, Germany

* Ulrike Krewer, ulrike.krewer@kit.edu

Keywords: Iridium oxide, catalyst degradation, reaction kinetics, surface coverage, electrocatalytic water splitting, dynamic modeling

Content

1 Computational Details 3

1.1 Model Implementation 3

1.2 Model Parameters 3

1.3 Parameter Identification Process 6

2 Additional Analysis 10

2.1 Electrochemical Degradation Results 10

2.2 CV Simulations with Different Scan Rates 13

2.3 Profile root mean square error analysis 14

2.4 Model Parameter Variation 16

2.5 Quantification of the Electroactive Surface Area (ECSA) 18

2.6 Oxidation State Validation 19

2.7 Logarithmic Charge-Current Relationship 21

2.8 Model Parameter Optimization of the Degrading System 22

1 Computational Details

1.1 Model Implementation

The microkinetic model is implemented and simulated in MATLAB 2020b. The set of differential equations is solved by applying an ode23s solver. High accuracy of the results is ensured by setting the absolute error tolerance to 10^{-12} and the maximum step size to 0.01 s.

1.2 Model Parameters

Parameter identification is crucial in order to gain a valid microkinetic model and, thus, valid model output, such as physically meaningful merits and insights into the dynamic behavior of the simulated system. For our study one can divide the parameters into three main categories: 1) Parameters of which the values are defined by the model assumptions e.g. the properties gained directly from the presumed mechanism itself. 2) Experimentally accessible parameter values, which can be measured easily or quantified by analyzing experimental data e.g. temperature and electrolyte resistance. 3) Parameters which are neither accessible directly with experimental methods nor defined by the model assumptions e.g. density of active sites or thermodynamic energies. These demand for either ab initio/DFT calculations or model-based identification algorithms to quantify their value from experiments. Unfortunately, DFT calculations show strong discrepancies in the resulting reaction energy values depending the computational details and assumptions[1], and they usually have high uncertainties, which in extreme cases may amount up to 0.66 eV. In addition, mostly reaction free energies were published so far, but few activation barrier energies, and to the best of our knowledge also no interaction energies of the adsorbed species. Hence, model-based parameter identification algorithms are required in order to completely parametrize the microkinetic model. In the following paragraphs, we will introduce the parameter identification process for all model parameters, which are needed to gain a valid model for dynamic simulations and further analyses.

The parameters of the first category are straightforward derived from the presumed mechanism. The matrix of the stochiometric coefficients ν_{ij} for all species $j \in \Omega = \{H^+; H_2O; O_2; e^-; *; *H_2O; *OH; *O; *OH_2O; *OOH; *OO\}$ are given for the forward reactions i as:

$$\boldsymbol{\nu}_+ = \left.\overbrace{\begin{pmatrix} 1 & 0 & 0 & 0 & 1 & 0 & 0 & 0 & 0 & 0 & 0 & 0 \\ 0 & 0 & 0 & 0 & 0 & 1 & 0 & 0 & 0 & 0 & 0 & 0 \\ 0 & 0 & 0 & 0 & 0 & 0 & 1 & 0 & 0 & 0 & 0 & 0 \\ 1 & 0 & 0 & 0 & 0 & 0 & 0 & 1 & 0 & 0 & 0 & 0 \\ 0 & 0 & 0 & 0 & 0 & 0 & 0 & 0 & 1 & 0 & 0 & 0 \\ 0 & 0 & 0 & 0 & 0 & 0 & 0 & 0 & 0 & 1 & 0 & 0 \\ 0 & 0 & 0 & 0 & 0 & 0 & 0 & 0 & 0 & 0 & 1 & 0 \\ 0 & 0 & 0 & 0 & 0 & 0 & 0 & 0 & 0 & 0 & 0 & 1 \end{pmatrix}}^{j \in \Omega}\right\} i \tag{S1}$$

and for the backward reactions as:

$$\boldsymbol{\nu}_- = \left.\overbrace{\begin{pmatrix} 0 & 0 & 0 & 0 & 0 & 1 & 0 & 0 & 0 & 0 & 0 & 0 \\ 0 & 1 & 0 & 1 & 0 & 0 & 1 & 0 & 0 & 0 & 0 & 0 \\ 0 & 1 & 0 & 1 & 0 & 0 & 0 & 1 & 0 & 0 & 0 & 0 \\ 0 & 0 & 0 & 0 & 0 & 0 & 0 & 0 & 1 & 0 & 0 & 0 \\ 0 & 1 & 0 & 1 & 0 & 0 & 0 & 0 & 0 & 1 & 0 & 0 \\ 0 & 1 & 0 & 1 & 0 & 0 & 0 & 0 & 0 & 0 & 1 & 0 \\ 0 & 0 & 0 & 0 & 0 & 0 & 0 & 0 & 0 & 0 & 0 & 1 \\ 0 & 0 & 1 & 0 & 1 & 0 & 0 & 0 & 0 & 0 & 0 & 0 \end{pmatrix}}^{j \in \Omega}\right\} i \tag{S2}$$

The parameters of the second category are quantified by the experimental properties and results. All experiments were conducted at a room temperature of $T = 25$ °C, and the geometrical electrode area was $A = 0.1963$ cm^2. The electrolyte resistance of $R = 18$ Ω was determined by electrochemical impedance spectroscopy measurements; the value was set to the impedance obtained at a phase angle of $\varphi = 0°$ in the high frequency range between $f = 10^5$ Hz and 10^4 Hz.

The activity of electrolyte species at the electrode interface was assumed to equal the respective bulk value due to fast transport compared to the consumption or production rates by the reactions. This assumption is reasonable, since in cyclic voltammetry experiments an increasing thickness of the Nernst diffusion layer accomplished by applying slower rotation speed indicated no significant limitation. Therefore, the activities were implemented as constant values of $a_{H_2O} = 1$ for water and $a_{H^+} = 0.2$ for protons, as the 0.1 M sulfuric acid is known to dissociate completely at low concentrations.

The parameters of the third category were quantified by the use of a parameter identification algorithm, which is described to full extend in the following subsection. Besides the density of active sites ρ and the double layer capacitance C_{dl}, this procedure was also applied to estimate the reaction free energy ΔG_{r} and activation free energy ΔG_{a} values of all seven reactions and the interaction free energy values of all seven adsorbed species ΔG_{int}.

It is noteworthy to state here, that in contrast to both the transition state theory, which is derived for forward reactions only, and the more generalised Marcus theory[2], we do not assume a pre-exponential frequency factor other than $k_0 = 343.2\ \mathrm{s}^{-1}$. This simplification is valid since we model the electrocatalytic system at constant temperature T and, hence, the exponential relation between the frequency factor k_0 and the activation energy ΔG_{a} makes it unfeasible to identify both parameters independently. For further explanation see the following section.

1.3 Parameter Identification Process

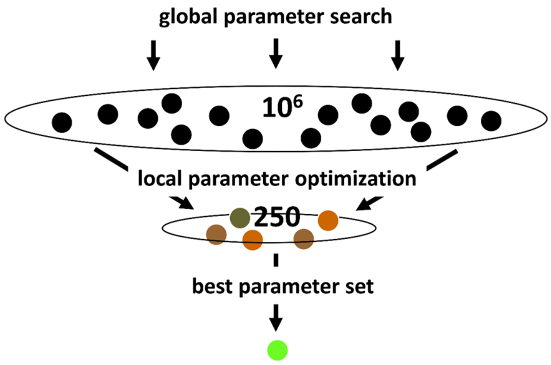

Figure S1: Schematic illustration of the two-step parameter identification process including the global parameter search of 10^6 randomly selected sets of parameters and the local parameter optimization of the best 250 sets.

To estimate the parameters of the third category, which are the reaction free energies, the activation free energies and the interaction free energies, the double layer capacitance and the density of active sites, we conducted an optimization procedure in two steps, as illustrated in **Figure S1** a). In a first step, the dynamic simulations were performed by randomly defining 10^6 sets of parameters within suitable ranges shown in **Table S1**. The ranges of the reaction free energy values of the electrochemical steps were set in such way that they were able to reproduce the features in the CV e.g. peak positions. For chemical steps, the ranges of the reaction free energies were selected in between wider limits, as they are not easily specifiable by the experimental current response. The reaction free energy of the oxygen evolution (step 7: *OO → * + O_2) is constrained by the thermodynamic formation energy $\sum \Delta G_{\mathrm{r}}^{0} = 4.92$ eV of the overall OER (2 H_2O → 2 H_2 + O_2). The preexponential frequency factor k_0 was set by the analysis of the peak-to-peak potential of anodic and cathodic redox transition, which converges to 0 V at a value of roughly $k_0 = 343.2\ \mathrm{s}^{-1}$ when assuming no activation barrier as shown in **Figure S2**. This value was chosen because we identify the activation free energy from the peak-to-peak potential. In consequence, the lower limits of the activation free energies were defined as ΔG_{a} = 0 eV. We assume as the upper range a value of 0.15 eV, since no significant peak to peak difference is observable in the CVs of the pristine material. One exception is the oxygen detachment (step 7: *OO → * + O_2)

which was found by DFT calculations to have an activation energy up to 0.58 eV[3, 4] and, thus, the respective activation free energy upper limit was increased to 1.65 eV. Lower and upper limits of the interaction energies of adsorbed species were set to 0 eV and 0.2 eV, respectively. The density of active sites and the double layer capacitance were found suitable to describe the experimental data in the ranges of [$8\cdot10^{-5}$, $20\cdot10^{-5}$] mol m^{-2} and [18, 20] F m^{-2} respectively.

Table S1: Parameter ranges for parameter identification using CVs

step	ΔG_r^0 / eV	ΔG_a / eV	ΔG_{int} / eV	ρ / mol m^{-2}	C_{dl} / F m^{-2}
(1)	[-3, 1]	[0, 0.15]	[0, 0.2]	[$8\cdot10^{-5}$, $20\cdot10^{-5}$]	[18, 20]
(2)	[0.7, 0.9]	[0, 0.15]	[0, 0.2]		
(3)	[1.0, 1.2]	[0, 0.15]	[0, 0.2]		
(4)	[-2, 2]	[0, 0.15]	[0, 0.2]		
(5)	[1.0, 1.4]	[0, 0.15]	[0, 0.2]		
(6)	[1.5, 2.0]	[0, 0.15]	[0, 0.2]		
(7)	$4.92 - \sum_{i\neq7} \Delta G_{r,i}^0$	[0, 1.65]	[0, 0.2]		

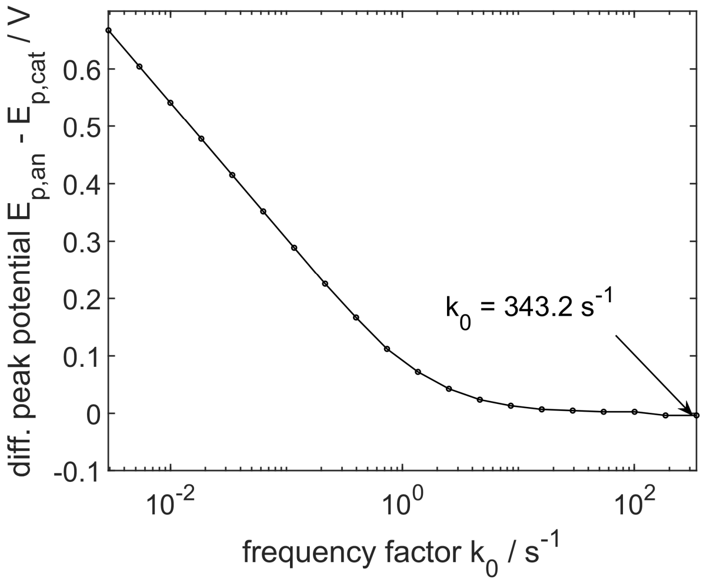

Figure S2: Simulated peak to peak position of a one-electron transfer reaction depends on the preexponential frequency factor k_0 with the activation free energy value set to 0 eV.

All 10^6 dynamic simulation results gained with the randomly selected sets of parameters were evaluated by comparing them to the experimental CV curves. As a quality measure we calculated the root mean square error (rmse) as given in equation (S3) including the total number $N = 1000$ of all simulated j and experimental j_{exp} current density values at $E > 0.5$ V:

$$rmse = \sqrt{\frac{1}{N}\sum_{n}(j - j_{\mathrm{exp}})^2} \tag{S3}$$

In a second step, the sets of parameters with the lowest rmse values were locally optimized by the use of a pattern search algorithm. This algorithm required higher computational costs and was, hence, only applied on the most promising 250 sets of parameters. The simulation data gained with the best optimized set matches the experimental data exceptionally well with a rmse value of only 0.066 mA cm^{-2}. The simulation results and the parameter values are shown in the main manuscript in **Figure 2 a)** and in **Table 1**, respectively.

The pattern search algorithm was also applied to identify the model parameter values of the steadily degrading catalytic system. Successively, the CV curves which were measured after each 30 minutes of operation at 1.6 V were used as input data j_{exp} in the objective function to minimize

the rmse. In every step, the values from the previously optimized set served as initial parameters. The simulation results as well as the rmse values are shown in the main manuscript in **Figure 7**.

2 Additional Analysis

2.1 Electrochemical Degradation Results

Application of a constant operating potential for a total duration of 15 hours in the acidic electrolyte leads to a decrease in the OER activity of the catalytic system. The dependence of changes in the CV on the constant potential is shown in **Figure S3** for 1.2 V, 1.5 V, 1.55 V and 1.6 V. The CV curves recorded each 150 minutes during the 15 hours constant potential protocol are given. Only minor absolute increase in redox transition current density and decrease of OER turnover frequency of 19 % is observable for the CV recorded in between an operation at a potential of 1.2 V in **Figure S3** a). Both observations are even more pronounced with higher applied potentials of 1.5 V in **Figure S3** b) and 1.55 V in **Figure S3** c). This reveals bigger loss in OER activity with higher applied potential, as indicated by the relative OER loss **Figure S3** d). It is noteworthy, that no decrease in the redox transition current is observed in the insets of the **Figure S3** a)-c), which indicates that in our case a loss of active material or the blockage by oxygen bubbles can be neglected. The EIS measurements at open circuit potential prior and after the protocol exhibit the electrolyte resistances to vary insignificantly with values changing from R = 19.2 Ω to 19.0 Ω, from R = 26.0 Ω to 24.8 Ω, from R = 20.0 Ω to 20.0 Ω and from R = 18.2 Ω to 18.4 Ω at the applied potentials of 1.2 V, 1.5 V, 1.55 V and 1.6 V respectively.

An additional experiment with constant potential of 1.6 V applied for 15 hours followed by a constant potential of 1.2 hours for 5 hours was conducted. The OER activity was measured by CV and is shown in **Figure S4**. After the performance decrease during operation at 1.6 V, a slight recovery of the activity is visible while applying a potential of 1.2 V afterwards.

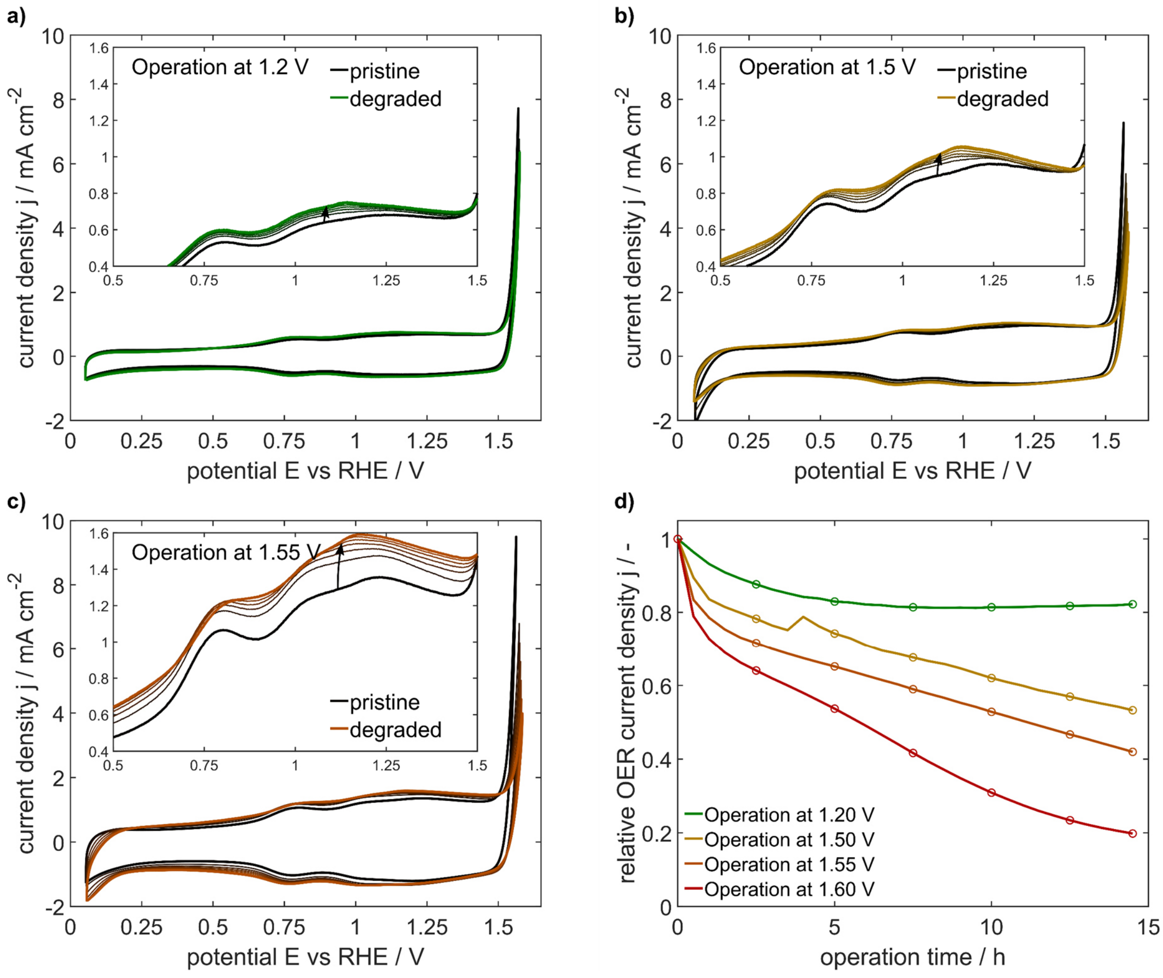

Figure S3: CV curves are recorded each 150 minutes during applying a constant potential of a) 1.2 V, b) 1.5 V and c) 1.55 V over 15 hours. The respective insets show the shift of the anodic redox transition with operating time. d) Relative change in current density at the highest applied potential during the CVs is shown over the operation time.

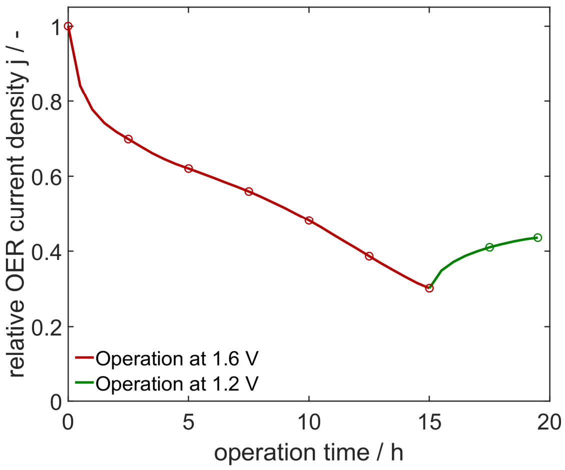

Figure S4: Change in relative current density of the CV curves during applying a constant potential of 1.6 V over 15 hours followed by a constant potential of 1.2 V over 5 hours.

2.2 CV Simulations with Different Scan Rates

Dynamic microkinetic simulations allow for arbitrary change of the model input, i.e. the applied potential over time. With the aim to prove that our model is able to reproduce the typical experimental variation in the potential scan rate, we show the simulated and the measured CV curves in **Figure S5** a) and b), respectively. In direct comparison the trend of higher absolute current density with several increasing scan rates starting from 25 mV/s up to 200 mV/s is valid for simulation and experiment in the lower potential range up to 1.45 V vs RHE. Both are in good agreement, which proves that the microkinetic model is able to reproduce this dynamic behavior.

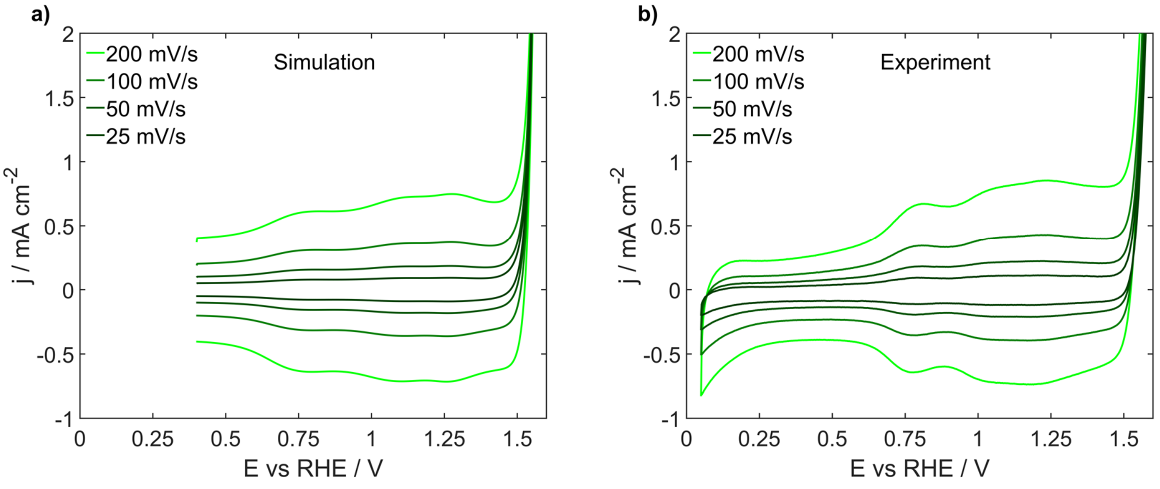

Figure S5: a) Simulated and b) experimental CV curves observed by applying different scan rates.

2.3 Profile root mean square error analysis

To ensure parameter identifiability of the reaction energy values, profile root mean square error (rmse) analysis was conducted. The method follows the profile likelihood analysis described elsewhere,[5] but using the rmse instead of the likelihood as a measure of deviation. To do so, parameters of the free reaction energy were successively variated from the identified value found by the method described in the SI in section 1.3 each by values of [(-0.2); -0.1; -0.05; 0; 0.05; 0.1; (0.2)] eV. Values in brackets are only used for $\Delta G_{r,4}$ and $\Delta G_{r,7}$. Then, the rmse was minimized by the pattern search algorithm, which was applied for all other parameters each with 25,000 objective function iterations.

In **Figure S6**, the resulting rmse values for variations of all reaction energy parameters and all optimized variations are given. Local minima in the profile rmse are observed, which indicates high identifiability of all analyzed parameters. As the minima in all profile rmse match with the rmse found for the identified model parameters, this analysis confirms that optimal reaction free energy values were identified.

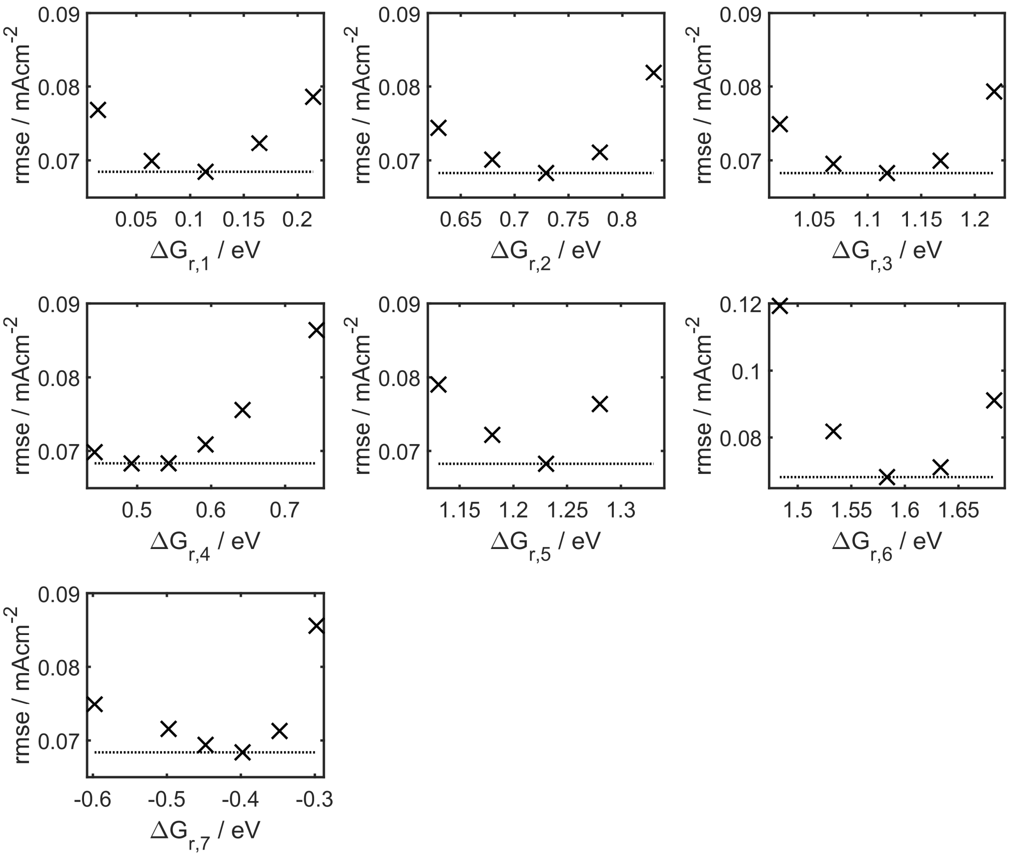

Figure S6: Root mean square error (rmse) profile analysis of the reaction free energy parameters of the OER mechanism on IrO_2. The dotted line indicates the lowest value gained by pattern search optimization.

2.4 Model Parameter Variation

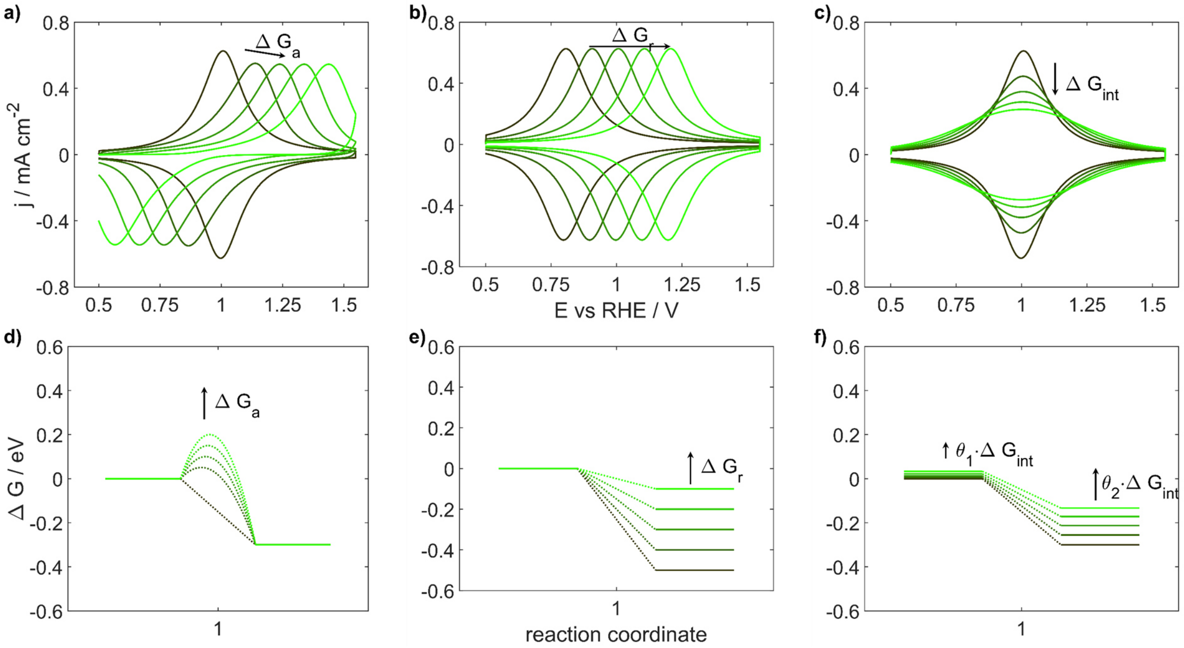

Figure S7: a-c) Simulated dynamic behavior of an elementary deprotonation step and d-f) respective energy diagrams at E = 1.3 V vs. RHE with variation of the activation free energy in a) and d), of the reaction free energy in b) and e) and of the interaction free energy in c) and f). Arrow directions indicate increase in denoted values. The parameters are given in **Table S2**.

To simulate the overall OER, a high amount of 23 parameters is needed to be identified. To visualize the effect of each energy parameter onto a single proton coupled electron transfer reaction, a simplified model of this one step is employed. CV simulations from E = 0.5 V to E = 1.5 V with a scan rate of 200 mV/s are shown in **Figure S7** a)-c), by the variation of the energy parameters given in **Table S2**. Bold numbers in the table define the values used to simulate the variation of the remaining parameters. The respective energy diagrams are shown in **Figure S7** d)-f). The increase of the activation free energy results in a shift of the peak maximum of the anodic current to higher potential and contrary to the shift of the peak minimum of the cathodic current to lower potential. High values of ΔG_a lead to an irreversibility of the transition. Higher reaction free energy values result in a shift of anodic and cathodic currents towards higher potential. The interaction free energy defines the broadening of the redox transition current response. The energies of reactant and product are increased proportional to the share of the surface coverage.

Hence, the analysis shows that for electrochemically driven elementary reactions, each energy parameter defines an identifiable feature in the dynamic current response.

Table S2: Values of the model parameter variation study with a single proton coupled electron transfer in **Figure S7**

variation	ΔG_{a} / eV	ΔG_{r} / eV	ΔG_{int} / eV	ρ / mol m^{-2}	C_{dl} / F m^{-2}
1	**0.00** [a]	0.8	**0.00** [a]	**10^{-4}** [a]	**0.025** [a]
2	0.20	0.9	0.05		
3	0.25	**1.0** [a]	0.10		
4	0.30	1.1	0.15		
5	0.35	1.2	0.20		

[a]Bold values of the varied parameters are held constant during variation of the remaining parameters

2.5 Quantification of the Electroactive Surface Area (ECSA)

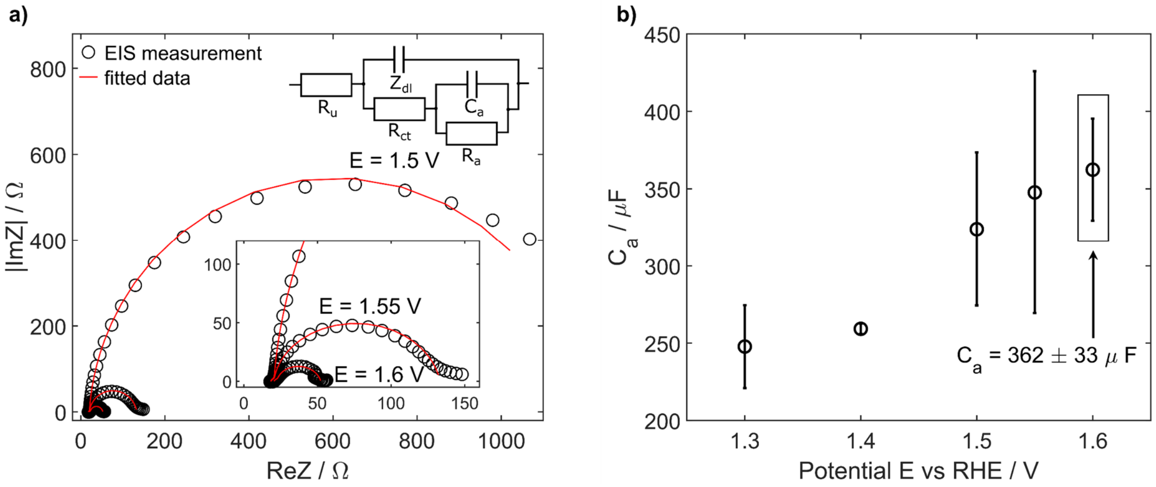

Figure S8: a) Measured impedance spectra and the best fit gained with the displayed equivalent circuit model are shown for three different potentials. b) Mean fitted values for the adsorption capacitance are given, with errors denoting the standard deviation of two independently analyzed measurements.

The electroactive surface area ECSA is quantified by using the method reported by the group of Bandarenka.[6] For this analysis, impedance spectroscopy measurements were conducted at potentials of 1.3 V, 1.4 V, 1.5 V, 1.55 V and 1.6 V with frequencies from $f = 10^5$ to 10^{-1} Hz and a perturbation amplitude of $E = 10$ mV. Selective results are shown in **Figure S8** a) and, following Bandarenkas publication, a simple equivalent circuit model, was used to reproduce the spectra and identify parameters.[6] Thus, the adsorption capacitance C_a was quantified as given in **Figure S8** b). It is the only parameter correlated to the ECSA. It shows the same increasing trend with increasing potential up to 1.6 V as reported for IrO_x.[6] To determine the ECSA from C_a, we used the procedure and value of the active-area specific adsorption capacitance C_a' from the Bandarenka group[6]. Here, the capacitance $C_{a,B}$ measured by EIS was normalized to the $ECSA_B$ of an IrO_x thin film determined by atomic force microscopy, which resulted in $C_a' = C_{a,B} / ECSA_B = 135 \pm 25$ µF cm^{-2}. This value is used to calculate the actual ECSA of the present system ECSA = $C_a / C_a' = 2.7 \pm 0.6$ cm^2.

2.6 Oxidation State Validation

The *in-situ* X-ray cell built for this analysis is based on the development of Binninger et al.[7] The cell preparation is explained to full extend in one of our previous reports from Czioska et al.[8] With a Gamry Reference 600+ potentiostat, constant potential steps of E = 0.6 V, 0.95 V, 1.15 V, 1.35 V, 1.5 V and 1.6 V vs RHE each of 20 minutes are applied for the duration of the x-ray absorption (XAS) measurements. *In-situ* XAS measurements were performed at Karlsruhe Institute of Technology synchrotron at the CATACT wiggler beamline using a Si(111) double-crystal monochromator. The XAS data were recorded at the Ir L_3-edge (11215 eV) in transmission mode. The raw data was then treated using Athena and Artemis.[9] The energies of the spectra were calibrated and aligned using reference metal foil channels to correct any energy shifts, and the spectra were normalized. The resulting position of the white line is shown in **Figure S9** and taken as an indicator for the change in Ir oxidation state. Although XAS radiation probes not only the active surface material but also the bulk material, a significant shift is observable with potential. Since the bulk material does not change significantly, the shift can be attributed to the Ir atoms on the outer surface. With the aim to compare the results to the model output we explain how we calculate the mean oxidation state in the following paragraph.

From the dynamically simulated coverages of adsorbed species, the mean oxidation state of the iridium active site is derived. Starting from the free iridium CUS * ≡ $Ir^{x+\delta}$ with the nominal oxidation state x and $\delta = 0$, the difference δ assigns the further influence due to the adsorption processes.[10] Therefore, for the following adsorbed species the difference in iridium oxidation state is given: *: $\delta = 0$, *H_2O: $\delta = 0$, *OH: $\delta = 1$, *O: $\delta = 2$, *OH_2O: $\delta = 2$, *OOH: $\delta = 1$, *OO: $\delta = 1$. The surface coverage, which corresponds to the share of surface covered by a species or its probability to be found at the surface, is multiplied with the difference in oxidation state. The results are shown in **Figure S9** for direct comparison to the operando XAS measurements.

In direct comparison to the white line peak position of the Ir L_3-edge absorption spectra at constant applied potentials, simulated and experimental results correspond on two main trends: 1) For both, an increase in the mean oxidation state with potential is observed in the range of 0.6 V to 1.35 V. 2) Both also show that at higher potentials, a maximum oxidation state is reached, after which the oxidation state decreases, thus, the active sites are reduced.

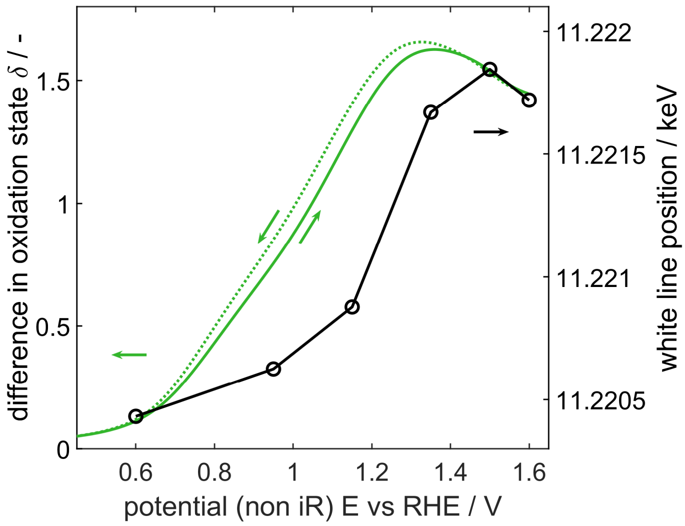

Figure S9: Potential-dependence of the simulated difference in mean oxidation state and the white line position of the absorption edge measured by *operando* XAS.

2.7 Logarithmic Charge-Current Relationship

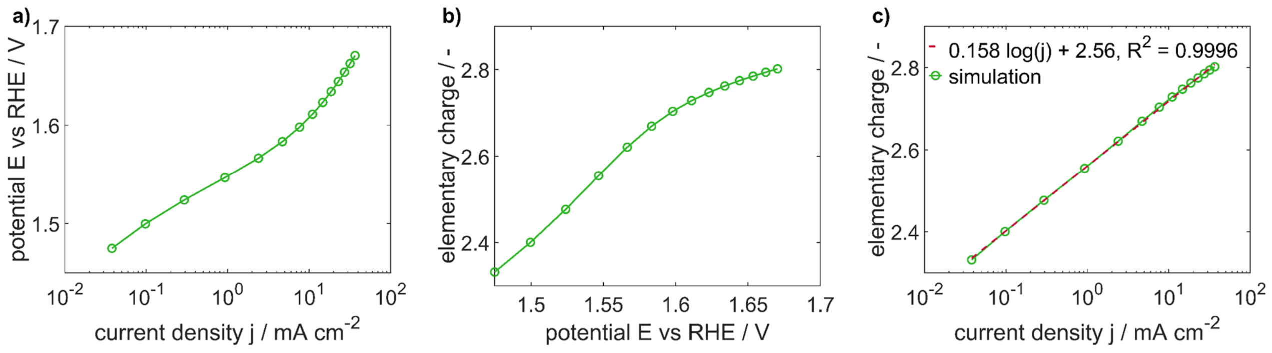

Figure S10: Relations between a) potential and current, b) number of elementary charges per active site and potential and c) number of elementary charges per active site and current are simulated with the microkinetic model.

The model prediction in the high OER potential regime is validated with the experimental polarization curve as shown in **Figure 6** in the main document. In addition, the elementary charge per active site can be easily evaluated by multiplying the coverage with the number of accumulated elementary charges of the respective adsorbed species. This merit is plotted in **Figure S10** versus the potential and the current. The logarithmic charge-current relation previously reported as a common relation of the OER[11] is perfectly reproduced by the simulation. Thus, we can conclude that the assumptions underlying the microkinetic model are valid for describing both basic correlations of the OER: The dynamic change in current with potential and the logarithmic steady-state relationship of elementary charge and current.

2.8 Model Parameter Optimization of the Degrading System

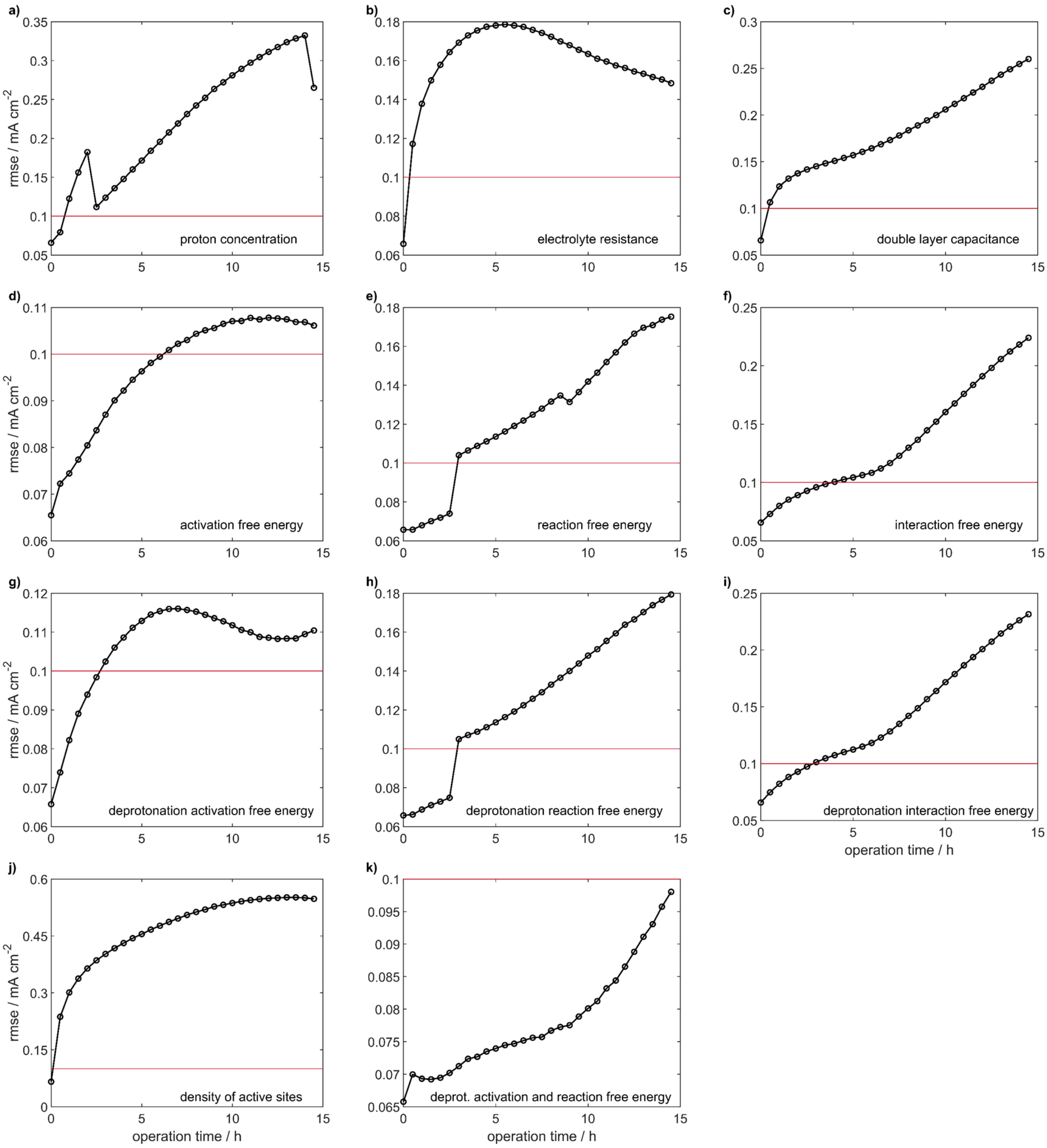

Figure S11: Resulting rmse estimator for the parametrization process of the CV curves during system degradation are evaluated with different optimized sets of parameters noted in the bottom right of each graph. The red line defines the threshold under which the best results are gained.

While optimizing the parameters during the steady degradation of the catalytic system, the root mean squared error (rmse) is tracked and given in **Figure S11**. The value of the density of active

sites is always optimized with parameter combinations tested as denoted in the bottom right of each graph to account for material dissolution, particle detachment and loss of binder material.

The change in proton concentration, electrolyte resistance and double layer capacitance are individually evaluated and the maximum rmse values of 0.33 mA cm^{-2}, 0.18 mA cm^{-2} and 0.26 mA cm^{-2} are gained respectively in **Figure S11** a-c). Although the change in electrolyte resistance is evaluated with lowest rmse so far, a maximum parameter value of R = 392 Ω is required to describe the completely degraded system, which is in contrast to the experimentally observed 18.2 Ω. Due to the high discrepancies we can disprove any correlation between the electrolyte-related parameters and the degradation behavior.

The free energy parameters are analyzed with the same method, and the results are shown in **Figure S11** d-i). Low values of the rmse and, thus, good reproduction of the experimental behavior is gained by optimizing all activation free energy values in **Figure S11** d). We found the activation free energy to be the best descriptor for the degradation process with a maximum rmse value not exceeding 0.11 mA cm^{-2}, which is significantly lower compared to the error values of 0.18 mA cm^{-2} for the reaction energy in **Figure S11** e) and 0.22 mA cm^{-2} for the interaction free energies in **Figure S11** f), respectively. Optimizing only activation free energies of the deprotonation steps hardly increase the rmse in **Figure S11** g). Also, the change of the reaction energy in **Figure S11** h) or the interaction energy in **Figure S11** i) of only deprotonation steps of gives comparable rmse values with a change in all steps. Evaluated with a high rmse value of up to 0.55 mA cm^{-2} in **Figure S11** j), the change of the density of active sites is not able to explain the experimentally observed degradation behavior solely. Best reproduction is obtained by the optimization of the activation and reaction free energies of the deprotonation steps, evaluated with a rmse remaining for each evaluated timestep below the 0.1 mA cm^{-2} threshold in **Figure S11** k). The simulation results and identified parameter values are shown in the main document in **Figure 7**.

REFERENCES

1. Briquet, L. G. V.; Sarwar, M.; Mugo, J.; Jones, G.; Calle-Vallejo, F., A New Type of Scaling Relations to Assess the Accuracy of Computational Predictions of Catalytic Activities Applied to the Oxygen Evolution Reaction. *ChemCatChem* **2017,** *9* (7), 1261-1268.

2. Marcus, R. A., On the Theory of Oxidation-Reduction Reactions Involving Electron Transfer. I. *Chem. Phys.* **1956,** *24* (5), 966-978.

3. Ping, Y.; Nielsen, R. J.; Goddard, W. A., 3rd, The Reaction Mechanism with Free Energy Barriers at Constant Potentials for the Oxygen Evolution Reaction at the IrO_2 (110) Surface. *J. Am. Chem. Soc.* **2017,** *139* (1), 149-155.

4. Zagalskaya, A.; Evazzade, I.; Alexandrov, V., Ab Initio Thermodynamics and Kinetics of the Lattice Oxygen Evolution Reaction in Iridium Oxides. *ACS Energy Lett.* **2021,** *6* (3), 1124-1133.

5. Cole, S. R.; Chu, H.; Greenland, S., Maximum likelihood, profile likelihood, and penalized likelihood: a primer. *Am. J. Epidemiol.* **2014,** *179* (2), 252-60.

6. Watzele, S.; Hauenstein, P.; Liang, Y.; Xue, S.; Fichtner, J.; Garlyyev, B.; Scieszka, D.; Claudel, F.; Maillard, F.; Bandarenka, A. S., Determination of Electroactive Surface Area of Ni-, Co-, Fe-, and Ir-Based Oxide Electrocatalysts. *ACS Catal.* **2019,** *9* (10), 9222-9230.

7. Binninger, T.; Fabbri, E.; Patru, A.; Garganourakis, M.; Han, J.; Abbott, D. F.; Sereda, O.; Kötz, R.; Menzel, A.; Nachtegaal, M.; Schmidt, T. J., Electrochemical Flow-Cell Setup for In Situ X-ray Investigations. *J. Electrochem. Soc.* **2016,** *163* (10), H906-H912.

8. Czioska, S.; Boubnov, A.; Escalera-López, D.; Geppert, J.; Zagalskaya, A.; Röse, P.; Saraçi, E.; Alexandrov, V.; Krewer, U.; Cherevko, S.; Grunwaldt, J.-D., Increased Ir–Ir Interaction in Iridium Oxide during the Oxygen Evolution Reaction at High Potentials Probed by Operando Spectroscopy. *ACS Catal.* **2021**, 10043-10057.

9. Ravel, B.; Newville, M., ATHENA, ARTEMIS, HEPHAESTUS: data analysis for X-ray absorption spectroscopy using IFEFFIT. *J. Synchrotron Radiat.* **2005,** *12* (Pt 4), 537-41.

10. Naito, T.; Shinagawa, T.; Nishimoto, T.; Takanabe, K., Recent advances in understanding oxygen evolution reaction mechanisms over iridium oxide. *Inorg. Chem. Front.* **2021,** *8* (11), 2900-2917.

11. Nong, H. N.; Falling, L. J.; Bergmann, A.; Klingenhof, M.; Tran, H. P.; Spori, C.; Mom, R.; Timoshenko, J.; Zichittella, G.; Knop-Gericke, A.; Piccinin, S.; Perez-Ramirez, J.;

Cuenya, B. R.; Schlögl, R.; Strasser, P.; Teschner, D.; Jones, T. E., Key role of chemistry versus bias in electrocatalytic oxygen evolution. *Nature* **2020,** *587* (7834), 408-413.
